# Seasonality, shelf life and storage atmosphere are main drivers of the microbiome and *E. coli* O157:H7 colonization of post-harvest lettuce cultivated in a major production area in California

**DOI:** 10.1186/s40793-021-00393-y

**Published:** 2021-12-20

**Authors:** Susan R. Leonard, Ivan Simko, Mark K. Mammel, Taylor K. S. Richter, Maria T. Brandl

**Affiliations:** 1 Office of Applied Research and Safety Assessment, Center for Food Safety and Applied Nutrition, U.S. Food and Drug Administration, Laurel, MD USA; 2 Crop Improvement and Protection Research Unit, US Department of Agriculture, Agricultural Research Service, Salinas, CA USA; 3 Produce Safety and Microbiology Research Unit, US Department of Agriculture, Agricultural Research Service, Albany, CA USA

**Keywords:** Produce, Leafy greens, Deterioration, Decay, Cultivar, Microbiota, MAP, Season, Enteric pathogen, STEC

## Abstract

**Background:**

Lettuce is linked to recurrent outbreaks of Shiga toxin-producing *Escherichia coli* (STEC) infections, the seasonality of which remains unresolved. Infections have occurred largely from processed lettuce, which undergoes substantial physiological changes during storage. We investigated the microbiome and STEC O157:H7 (EcO157) colonization of fresh-cut lettuce of two cultivars with long and short shelf life harvested in the spring and fall in California and stored in modified atmosphere packaging (MAP) at cold and warm temperatures.

**Results:**

Inoculated EcO157 declined significantly less on the cold-stored cultivar with short shelf life, while multiplying rapidly at 24 °C independently of cultivar. Metagenomic sequencing of the lettuce microbiome revealed that the pre-storage bacterial community was variable but dominated by species in the *Erwiniaceae* and *Pseudomonadaceae*. After cold storage, the microbiome composition differed between cultivars, with a greater relative abundance (RA) of *Erwiniaceae* and *Yersiniaceae* on the cultivar with short shelf life. Storage at 24 °C shifted the microbiome to higher RAs of *Erwiniaceae* and *Enterobacteriaceae* and lower RA of *Pseudomonadaceae* compared with 6 °C. Fall harvest followed by lettuce deterioration were identified by recursive partitioning as important factors associated with high EcO157 survival at 6 °C, whereas elevated package CO_2_ levels correlated with high EcO157 multiplication at 24 °C. EcO157 population change correlated with the lettuce microbiome during 6 °C storage, with fall microbiomes supporting the greatest EcO157 survival on both cultivars. Fall and spring microbiomes differed before and during storage at both temperatures. High representation of *Pantoea agglomerans* was a predictor of fall microbiomes, lettuce deterioration, and enhanced EcO157 survival at 6 °C. In contrast, higher RAs of *Erwinia persicina, Rahnella aquatilis*, and *Serratia liquefaciens* were biomarkers of spring microbiomes and lower EcO157 survival.

**Conclusions:**

The microbiome of processed MAP lettuce evolves extensively during storage. Under temperature abuse, high CO_2_ promotes a lettuce microbiome enriched in taxa with anaerobic capability and EcO157 multiplication. In cold storage, our results strongly support a role for season and lettuce deterioration in EcO157 survival and microbiome composition, suggesting that the physiology and microbiomes of fall- and spring-harvested lettuce may contribute to the seasonality of STEC outbreaks associated with lettuce grown in coastal California.

**Supplementary Information:**

The online version contains supplementary material available at 10.1186/s40793-021-00393-y.

## Background

Plants are hosts to diverse microbial communities that are critical to their health. Still unexplored is the role of the plant microbiome in human health, and particularly its effect on the microbial safety of the many plant-derived foods that are part of the human diet [1–3]. *Proteobacteria* are the dominant plant colonists of most plant species investigated to date, along with *Actinobacteria*, *Bacteroidetes* and *Firmicutes* [4]. Although *Enterobacteriaceae* are not as common as other members of the Proteobacteria on plants, they are frequent constituents of plant microbiota and occasionally may be detected in high relative abundance [5–9]. Many *Enterobacteriaceae* species straddle hosts, but they primarily colonize animals, including humans [10–13]. The presence of zoonotic human pathogens on crops, many of which belong to the *Enterobacteriaceae* is among the most important threats to public health and the horticultural industry since fruit and vegetables became the primary vehicle of foodborne illness in the USA and other countries at the turn of the century [14–16]. In 2013, the European Food safety Authority listed fresh fruit and vegetables, including leafy greens, on the highest priority list for prevention of outbreaks and control measures due to major public health concerns [17].

Shiga toxin-producing *Escherichia coli* (STEC), in particular serotype O157:H7 (EcO157), is a main etiological agent of foodborne disease outbreaks linked to the consumption of produce, especially leafy vegetables [14, 16, 18]. While STEC is prevalent in the environment in produce-growing regions of the USA [19–21], it is detected only sporadically and at low levels on lettuce in the field and at retail [22–24]. EcO157 displays weak fitness as a plant colonist in field studies [25–27], although it has the ability to multiply in the lettuce phyllosphere under growth-permissive conditions in the laboratory [28, 29]. Despite this low prevalence and poor fitness on lettuce crops, EcO157 has caused numerous outbreaks of disease attributed to lettuce in the USA, Canada and Europe [16, 18, 30–32]. Additionally, lettuce-associated outbreaks of STEC in the USA follow a seasonal pattern as infections occur more frequently from lettuce harvested in the fall on the West coast of California and in the late winter-early spring in the Southeastern part of California and in Arizona [33, 34].

Outbreaks of EcO157 infections due to contaminated lettuce in the USA have been linked largely to fresh-cut product. In laboratory studies, EcO157 multiplied rapidly in cut lettuce under conditions simulating temperature abuse [35]. Plant lesions are inherent to harvesting and fresh-cut processing. Similar to colonization of wounds in humans by opportunistic pathogens that are common members of the plant microbiota [36], plant injury provides new opportunities for zoonotic pathogens to colonize an otherwise less than hospitable habitat. Our previous transcriptome studies revealed the numerous pathways through which enteric pathogens adapt to, and benefit from, leaf lesions [37, 38].

Enteric pathogen behavior in plant wounds is not governed solely by the alteration of the plant physicochemical environment due to injury but may be influenced further by conditions imposed through storage of the cut product and by its microbiota. The increased respiration and transpiration rates of cut leaf tissue hasten its natural deterioration by senescence and shorten the shelf life of minimally processed lettuce. The presence of microbes with the ability to enzymatically macerate the plant cell wall may also contribute to decay. Hence, cold storage under controlled atmosphere that reduces the respiration rate of the plant tissue and certain spoilage organisms is critical to extend the shelf life of cut lettuce [39]. In this approach, the ambient atmosphere in the package is displaced by one limited in oxygen concentration, which is maintained by using a polymer film with low oxygen transfer rate. Loss of shelf life is manifested by the occurrence of limp, water-logged tissue and its gradual liquefaction due to cell lysis. This type of deterioration, which appears to be associated with plant physiological processes in modified atmosphere packaging (MAP), is a genetically traceable trait and lettuce accessions with greatly different rates of deterioration after fresh-cut processing have been identified [40]. While lettuce deterioration in MAP can occur under cold storage, it is greatly hastened under warm temperature. Maintenance of the old chain of fresh-cut produce slows tissue degradation and is also critical to the microbial safety of the product. Nevertheless, warm temperature abuse occurs frequently once perishable food enters the distribution chain, thereby jeopardizing its quality and safety [41]. Thus, fruit and vegetables are among the foods most investigated for time–temperature management [42].

Given the high incidence of human infections of STEC associated with fresh-cut lettuce packaged under modified atmosphere and the inherent short shelf life of this product, it is critical to public health to improve our knowledge of the microbial, physicochemical, and plant factors that contribute to this well-established trend. In the study described herein, we sought to determine the role of MAP lettuce shelf life in EcO157 survival during cold storage and multiplication under temperature abuse using cultivars with different deterioration rates harvested during the spring and fall seasons in Salinas, CA, the largest lettuce production region of the USA. Furthermore, we extensively characterized the dynamic evolution of the lettuce microbiome in this context and that of atmospheric conditions developing as a result of the plant physiological adaptation to injury and MAP in order to gain a holistic understanding of the behavior of STEC in this post-harvest environment.

## Results

**Fig. 1:**
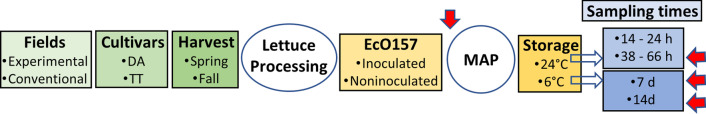
Schematic representation of the study workflow and the variables included in the experiments, illustrated by boxes. Red arrows depict the points of sampling for characterization of microbiomes on the processed lettuce prior and after storage

We investigated the colonization of minimally processed romaine lettuce by EcO157 over time during storage in MAP at cold and warm temperature in the context of lettuce shelf life and of the bacterial communities that reside on the lettuce. The microbiomes of noninoculated MAP lettuce samples were also examined. Lettuce cultivars with long shelf life (cultivar DA) and short shelf life (cultivar TT) were planted in Salinas, CA, in a commercial lettuce field under conventional cultivation and in an experimental field. The lettuce was harvested in the spring and the fall over the course of five experiments. Sampling for EcO157 population size quantification and/or microbiome characterization was conducted after lettuce processing (prior to storage) and at mid- and endpoint of storage. Figure 1 depicts a general outline of our study. Further details are provided below in “Methods”.

### Post-harvest lettuce core microbiomes

Post-harvest core microbiomes, which represent the expected taxa on processed romaine lettuce prior to and during storage, independent of cultivar, season, or field, were determined after removing EcO157 when the pathogen was present. We were interested in determining the lettuce microbiomes as they naturally exist without the inoculated pathogen, thus removed EcO157 to compute relative abundances of the naturally existing taxa in this analysis, and others as stated in the text. The core microbiome following lettuce processing and prior to MAP storage was small, accounting for only 25% of the total bacterial community, and was composed of *Pseudomonas fluorescens* (9%), *Pantoea agglomerans* (15%), and other *Pantoea* species (2%) (Table 1). Thus, noncore taxa represented 74% of the total bacterial community, indicating a high variability among all processed samples before storage. This suggests that the bacterial communities on the leaf tissue were not completely homogenized during washing and that the chlorinated wash in our study may have effectively reduced cross-contamination. MAP storage increased the core microbiome to a similar level of 68% at both 6 °C (mid- and endpoint samples combined) and 24 °C (endpoint). At 6 °C, the core lettuce microbiome was equally represented by the *Erwiniaceae* and *Pseudomonadaceae* (34%), demonstrating that members of these families displaced noncore taxa in representation. The Pseudomonas species diversified during storage at both 6 °C and 24 °C, however, in contrast to 6 °C storage, the *Pseudomonadaceae* proportionally decreased in RA in the core microbiome during 24 °C storage. The expansion of the core microbiome under temperature abuse was thus largely due to an increase in RA as well as species diversification of the *Erwiniaceae* (41%), and a high proportion of *Enterobacteriaceae* (19%), in particular, *Enterobacter cloacae* and *Leclercia adecarboxylata*.

**Table 1:** Core microbiomes of lettuce after processing and prior to storage, and during MAP storage

	Taxa	Relative abundance (%)	
Pre-storage	*Erwiniaceae Pantoea*	1.77	16.53
	*Erwiniaceae Pantoea agglomerans*	14.76	
	*Pseudomonadaceae Pseudomonas fluorescens*	8.92	8.92
	Noncore taxa	*74.56*	
6 °C storage	*Erwiniaceae*	1.53	33.96
	*Erwiniaceae Pantoea*	3.25	
	*Erwiniaceae Pantoea agglomerans*	29.17	
	*Pseudomonadaceae Pseudomonas*	0.51	34.13
	*Pseudomonadaceae Pseudomonas moraviensis*	14.37	
	*Pseudomonadaceae Pseudomonas fluorescens*	10.38	
	*Pseudomonadaceae Pseudomonas koreensis*	7.91	
	*Pseudomonadaceae Pseudomonas granadensis*	0.95	
	Noncore taxa	*31.91*	
24 °C storage	*Erwiniaceae*	2.07	40.59
	*Erwiniaceae Pantoea*	4.24	
	*Erwiniaceae Pantoea agglomerans*	32.25	
	*Erwiniaceae Pantoea vagans*	0.12	
	*Erwiniaceae Erwinia*	0.86	
	*Erwiniaceae Erwinia persicina*	0.84	
	*Erwiniaceae Erwinia gerundensis*	0.20	
	*Enterobacteriaceae*	1.22	19.33
	*Enterobacteriaceae Enterobacter cloacae*	11.49	
	*Enterobacteriaceae Leclercia adecarboxylata*	6.61	
	*Pseudomonadaceae Pseudomonas moraviensis*	3.92	7.57
	*Pseudomonadaceae Pseudomonas koreensis*	2.05	
	*Pseudomonadaceae Pseudomonas fluorescens*	1.60	
	Noncore taxa	*32.52*	

Core microbiomes were determined based on all DA and TT samples, inoculated and noninoculated

### Effect of lettuce genotype on deterioration and microbiome

**Fig. 2:**
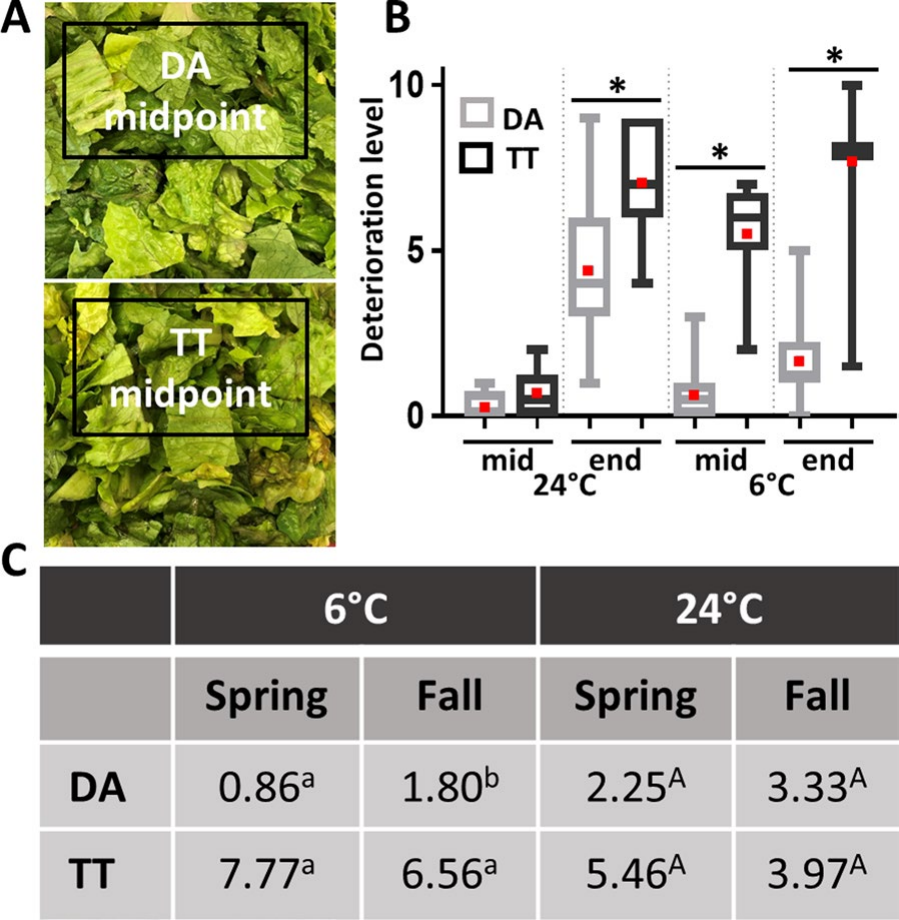
Shelf life in lettuce cultivars DA and TT under MAP storage conditions. **a** Representative images of fresh-cut lettuce of each cultivar at midpoint of storage at 6 °C. **b** Box plots of deterioration levels on a scale of 0–10 displayed by both cultivars at mid- and endpoint of storage at 24 °C and 6 °C. Data represent all samples across five experiments including different locations and seasons. Red dots and * represent means and significant difference in the means between cultivar DA and TT at a given sampling point (24 °C: Student’s *t* test, *P* < 0.0001; 6 °C: Mann–Whitney test, exact *P* < 0.0001). **c** Table of mean decay ratings in cultivars DA and TT per harvest season under each storage temperature. Values represent the average for all samples at mid- and endpoint of storage. Different letters under a given temperature indicate a significant difference between spring and fall lettuce within a given cultivar (Mann–Whitney test with exact *P* value on Logit-transformed decay ratings, with values of zero replaced with 0.01 before transformation; *P* < 0.05)

*Deterioration* Romaine lettuce cultivars DA and TT differed significantly in shelf life at mid- and endpoint of storage in MAP at 6 °C as evidenced by a large percentage of leaves displaying loss of integrity in TT compared with DA (Fig. 2a). TT leaves had mean deterioration ratings 4.5- and 6.8-fold greater than DA leaves after ca. 7 and 14 days of cold storage, respectively (Mann–Whitney test; exact *P* = 0.0001) (Fig. 2b). At 24 °C, deterioration ratings differed significantly between the two cultivars only at end point of storage (ca. 2 days) (Fig. 2b; Student’s *t* test; *P* = 0.0001), despite a visible onset of deterioration in TT but not DA after only 1 day of storage. Furthermore, deterioration ratings in DA stored at 6 °C were significantly greater in the fall- than in the spring-harvested lettuce (Mann–Whitney test; exact *P* < 0.05), unlike in TT (Fig. 2c). Nevertheless, deterioration in fall-harvested DA lettuce was on average much lower than that observed in TT. The difference in rating values between spring- and fall-harvested lettuce stored at 24 °C were not statistically significant (Mann–Whitney test; exact *P* = 0.28 for DA, exact *P* = 0.08 for TT).

*Taxa with decay capability in the microbiomes*. Several taxa with reported potential to actively decay post-harvest plant tissue were identified in the microbiomes of lettuce prior to, and during storage at 6 °C and 24 °C. These included members of the *Pectobacteriaceae* (*Dickeya dianthicola*, *D. solani*, *Pectobacterium carotovorum*); *Pseudomonadaceae* (*Pseudomonas marginalis*, *P. viridiflava*, *P. wasabiae*); *Leuconostocaceae* (*Leuconostoc mesenteroides*, *L. pseudomesenteroides*, *L. kimchii*); *Enterobacteriaceae* (*Klebsiella variicola*); and *Bacillaceae* (*Bacillus altitudinis*) (Additional file 1: Table S1). Before storage, most of the above species represented less than 1–2% of the total bacterial community and were not detected in the majority of the samples (Additional file 1: Table S1a). *P. marginalis* was undetected or present at low RA in most 6 °C-stored samples but was present at RA > 10% in 10/51 samples from the conventional field, in particular in TT lettuce in spring 2018 but also in DA lettuce in both spring and fall 2018 (Additional file 1: Table S1b). Likewise, *P. viridiflava* was detected in a subset of 6 °C microbiomes, independent of season and field/cultivation type, but only in DA and at a low RA that did not exceed 7% in any sample. While the few bags of TT lettuce with exceptionally high *P. marginalis* also had high levels of deterioration, other bags of 6 °C-stored TT exhibited similar levels of deterioration but had either undetectable or very low levels of this pathogen. Likewise, the presence of the two soft rot pathogens in DA did not de facto result in extensive deterioration. *L. mesenteroides*, a spoilage species common in cold-stored foods was present at noticeable RA (< 7%) in a few samples of TT lettuce originating from the experimental and conventional fields.

After storage at 24 °C, only six samples of TT lettuce from the conventional field in spring 2018 harbored *P. marginalis,* while *P. viridiflava* was not detected in any sample (Additional file 1: Table S1c). *L. mesenteroides* was present in several samples of DA and TT from the conventional field with a RA ≥ 1%. The well-characterized members of the *Pectobacteriaceae* family of soft rot pathogens were rarely present at 24 °C and had a very low RA of less than 5%. Overall, the lettuce microbiomes during 6 °C storage harbored the broadest diversity of taxa with a potential role in plant tissue decay and with the greatest representation compared with warm storage.

**Fig. 3:**
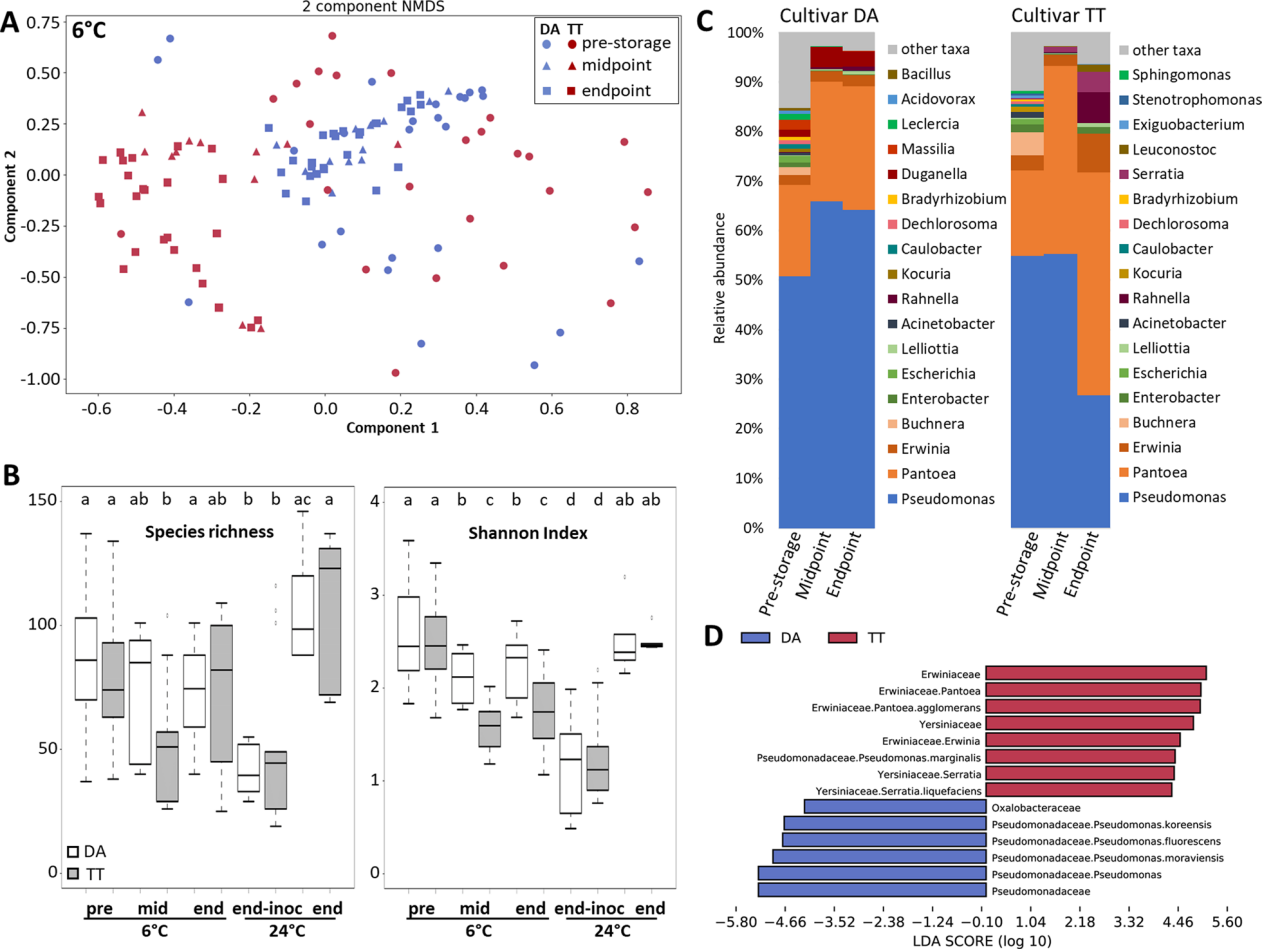
Romaine lettuce microbiome progression during storage under MAP conditions. **a** NMDS plot of bacterial community structure on processed lettuce of cultivars DA and TT before and at mid- and endpoint during storage at 6 °C. **b** Comparison of alpha diversity including species richness and Shannon index between cultivars DA and TT across time points during storage at 6 °C and 24 °C. The bottom and top edges of the boxes mark the 25th and 75th percentiles, and the horizontal line denotes the median. Letters indicate significant differences (Wilcoxon rank sum test with Benjamini–Hochberg adjustment, *P* < 0.05). The inoculated EcO157 contributed significantly to the bacterial community with 24 °C storage (but not 6 °C storage) thus alpha diversity indexes are depicted both with and without the EcO157 included in determining bacterial community structure for 24 °C storage. **c** Average relative abundance (RA) of bacterial genera in lettuce metagenomes prior and during storage at 6 °C. Genera with RA ≥ 0.4 for at least one time point are listed and identified taxa with lower RA are included in ‘other taxa’. **d** Histogram of LDA scores computed using LEfSe analysis for taxa differentially abundant, including all five harvests, between the metagenomes of cultivars DA and TT after storage at 6 °C (biomarkers with LDA value ≥ 4.2 depicted)

*Microbiome progression during cold storage differs by cultivar*. Bacterial communities associated with the processed lettuce before storage and at the two sampling times during cold storage were determined. Beta diversity analysis revealed that the bacterial communities progressed during 6 °C storage from a broad heterogenous distribution independent of lettuce cultivar at the start of storage (PERMANOVA, *P* = 0.0608) to a tight cluster of microbiomes in cultivar DA that were different from those in cultivar TT (Fig. 3a and Additional file 2: Table S2). Whereas DA bacterial communities were very similar at mid- and endpoint, and several TT microbiomes clustered with DA microbiomes at midpoint, the TT and DA microbiomes were significantly different at both storage times (PERMANOVA; *P* = 0.0001 for both mid and endpoint) (Additional file 2: Table S2). Alpha diversity, as measured by the species richness and Shannon index, was highly variable across samples for both the DA and TT pre-storage microbiomes and did not differ between the two cultivars (Fig. 3b). Species richness, which remained highly variable for lettuce stored at 6 °C, was unchanged in DA microbiomes throughout storage, but decreased at midpoint for TT. For both cultivars, the Shannon index decreased by midpoint and then remained unchanged, with bacterial communities associated with TT exhibiting a greater decline.

The pre-storage lettuce microbiomes on both cultivars were primarily composed of *Pseudomonas* with RA 50–55% and *Pantoea* and *Erwinia*, both members of the *Erwiniaceae* with a combined RA of 20–21% (Fig. 3c). The individual sample microbiomes included other species within those genera, as well as other genera, as is consistent with the wide variation in alpha diversity (Fig. 3b) and high RA of non-core taxa (Table 1). *Enterobacteriaceae* constituted the third most prevalent family, including *Enterobacter*, *Escherichia* (predominantly *albertii*), *Lelliottia*, and *Leclercia*. Overall, the bacterial communities associated with DA and TT were not statistically different before storage (after processing). However, variation in low abundant taxa was observed in their average microbiomes when examined separately (Fig. 3c).

Over the 2 week-storage period at 6 °C, the RA of *Pseudomonas* on DA increased to at least 64%, while that of the *Erwiniaceae* (*Pantoea, Erwinia*, and *Buchnera*) remained relatively stable (Fig. 3c). On the contrary, the relative abundance of *Pseudomonas* on TT decreased to 27% at endpoint, while that of the *Erwiniaceae* increased steadily to reach 53%. The differential RAs of *Pseudomonas* and *Erwiniaceae* explained a large part of the shifts in microbiome structure in the two cultivars under cold storage. Of note also, is the presence of *Leuconostoc* in the endpoint microbiomes on cultivar TT (Fig. 3c), the leaves of which showed considerable deterioration (Fig. 2). On average, the RA of *Enterobacteriaceae* on DA at the end of storage was lower than on TT, with values of 1% and 3%, respectively. The only Enterobacteriaceae genera with RA above 0.05% in the DA endpoint microbiomes were *Enterobacter* and *Lelliottia*, while TT endpoint microbiomes also included greater than 0.05% RA of *Cedecea, Citrobacter, Kluyvera, Kosakonia, Leclercia,* and *Klebsiella.*

Consistent with the divergent shifts in microbiome structure in the two cultivars, the linear discriminant effect size tool (LEfSe) [43] identified various *Pseudomonas* specie*s* as enriched in DA microbiomes at 6 °C, with the exception of the soft rot pathogen *P. marginalis*, which was identified as enriched on average in TT microbiomes (Fig. 3d and Additional file 3: Fig. S1). In contrast, *Pantoea* and *Erwinia* were identified as significantly enriched in TT microbiomes. Additional file 3: Fig. S1 provides details of additional biomarkers in cold-stored DA and TT.

*Microbiome shift is independent of cultivar under temperature abuse*. Unlike the results for lettuce stored at 6 °C, bacterial communities associated with lettuce stored at 24 °C did not differ significantly between DA and TT at endpoint (PERMANOVA, *P* = 0.1862) (Additional file 4: Fig. S2a). Storage at 24 °C resulted in a shift in microbiome composition to a higher *Erwinaceae*/*Pseudomonadaceae* ratio (Additional file 4: Fig. S2b), as was observed for the TT cultivar during 6 °C storage (Fig. 3c). Importantly, on average the RA of *Enterobacteriaceae* expanded to approximately 30% (excluding the inoculated EcO157 in the analysis) in contrast to cold stored lettuce where this family remained a minor component (< 2%) of the microbiome (Fig. 3c and Additional file 4: Fig. S2b).

### Effect of EcO157 inoculation on the lettuce microbiome

Inoculation of lettuce with EcO157 did not affect the DA or TT microbiomes under cold storage; the human pathogen population decreased over time after inoculation to less than 0.07% relative abundance in eight inoculated samples and remained undetected in the microbiome of the remaining 38 inoculated samples (data not shown). However, examination of the microbiomes associated with inoculated lettuce stored at 24 °C, from which EcO157 was removed for analysis, revealed a significant difference from that of noninoculated samples (PERMANOVA, *P* = 0.0205), indicating that the inoculated human pathogen had an effect on the lettuce microbiome under temperature abuse (Additional file 4: Fig. S2c). Additionally, both the Shannon index and species richness were reduced significantly in the EcO157-inoculated lettuce stored at 24 °C but did not change in noninoculated lettuce bacterial communities (Fig. 3b). On average, inoculation resulted in an increase in RA of *Pseudomonas* of approximately 2% with a concomitant decrease in *Serratia* (Additional file 4: Fig. S2b). While the overall relative abundance of *Enterobaceriaceae* remained almost unchanged, inoculation resulted in shifts in the family composition with substantial decreases in *Leclercia* and *Kosakonia* and an increase in *Enterobacter*.

### Correlation of EcO157 colonization, MAP atmosphere, and deterioration with lettuce microbiome composition

**Fig. 4:**
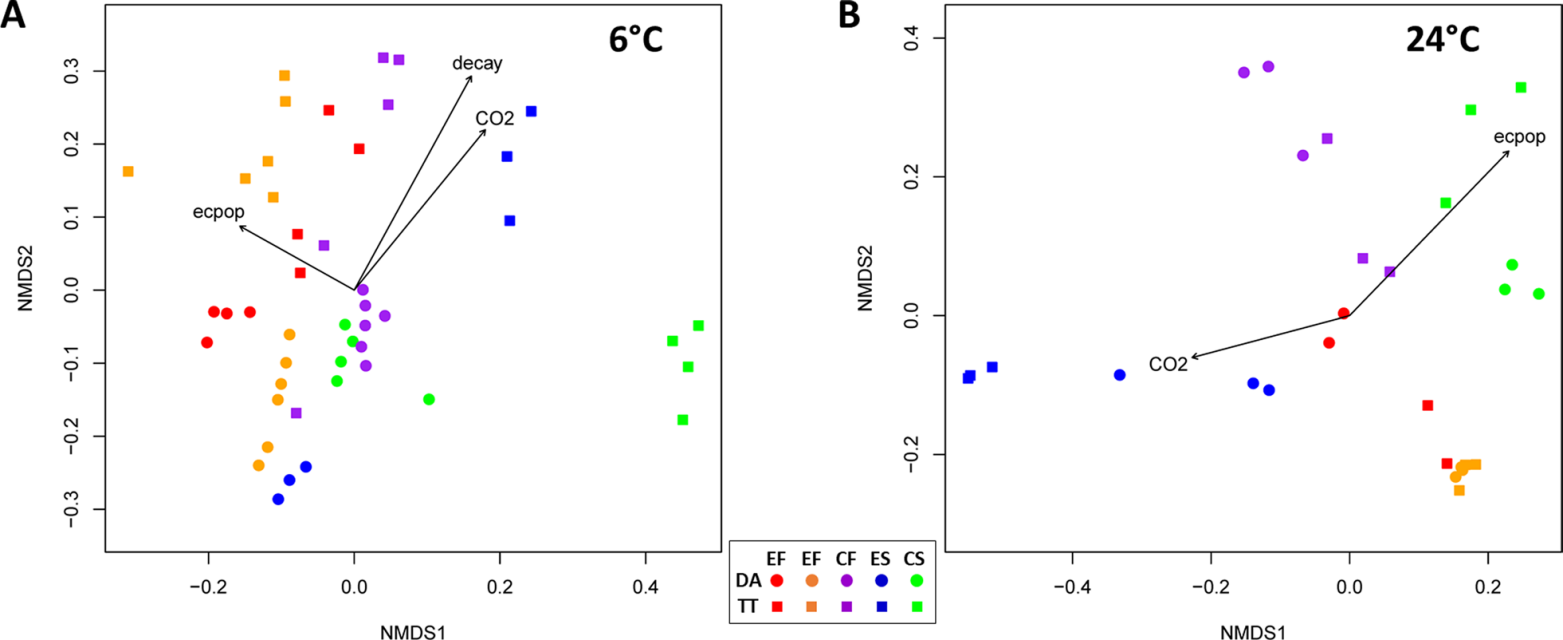
Correlations between bacterial community composition on lettuce sampled at mid- and endpoint of storage under MAP conditions and various factors. Factors included in the analysis were decay rating, %CO_2_ and %O_2_ in the bag, and difference between inoculated EcO157 population size prior to storage and at either mid- or endpoint (ecpop). Only inoculated samples are included. NMDS beta diversity plots include cultivars DA and TT for the five harvests including experimental field (E), conventional field (C), fall (F), and spring (S) and colored as denoted in the legend. Vectors were computed using EnvFit and only those with *P* < 0.05 are included in the plots. **a** Storage at 6 °C. Significant correlations were observed for decay (R^2^ = 0.756, *P* = 0.0001), %CO_2_ (R^2^ = 0.546, *P* = 0.0001), and ecpop (R^2^ = 0.218, *P* = 0.0053). **b** Storage at 24 °C. Significant correlations were observed for %CO_2_ (R^2^ = 0.326, *P* = 0.0075), and ecpop (R^2^ = 0.638, *P* = 0.0001). Correlation between the lettuce metagenomes and %O_2_ in the bag was not significant for either storage temperature

The envfit function was used to determine relationships at mid- and endpoint of storage between the bacterial community composition and variables of interest: MAP atmosphere, lettuce deterioration, and EcO157 population change (Fig. 4). In order to include the change in EcO157 population size on the lettuce as a covariate, only microbiome data from inoculated lettuce samples were used in the analysis. The association between variables tested and bacterial communities on lettuce stored at 6 °C was strongest for deterioration rating (*P* = 0.0001, R^2^ = 0.76) and %CO_2_ (*P* = 0.0001, R^2^ = 0.55); fitted vectors pointed toward TT microbiomes (Fig. 4a). The change in Log-value of EcO157 population size was significantly but weakly correlated with the lettuce microbiome during cold storage (*P* = 0.0053, R^2^ = 0.22) and the vector pointed toward fall microbiomes. In contrast, change in EcO157 population size was the variable with the strongest correlation with microbiome composition during storage at 24 °C (*P* = 0.0001, R^2^ = 0.64). %CO_2_ was also a significant covariate (*P* = 0.0076, R^2^ = 0.33), while no correlation was observed with deterioration level (Fig. 4b). No significant correlation was observed between O_2_ and bacterial community composition at either storage temperature. Fitted vector direction did not generally align with either cultivar for microbiomes of lettuce stored at warm temperature, supporting our observations that lettuce cultivar did not have a significant effect on most variables that we measured under temperature abuse.

### Effect of lettuce shelf life on EcO157 population dynamics

**Fig. 5:**
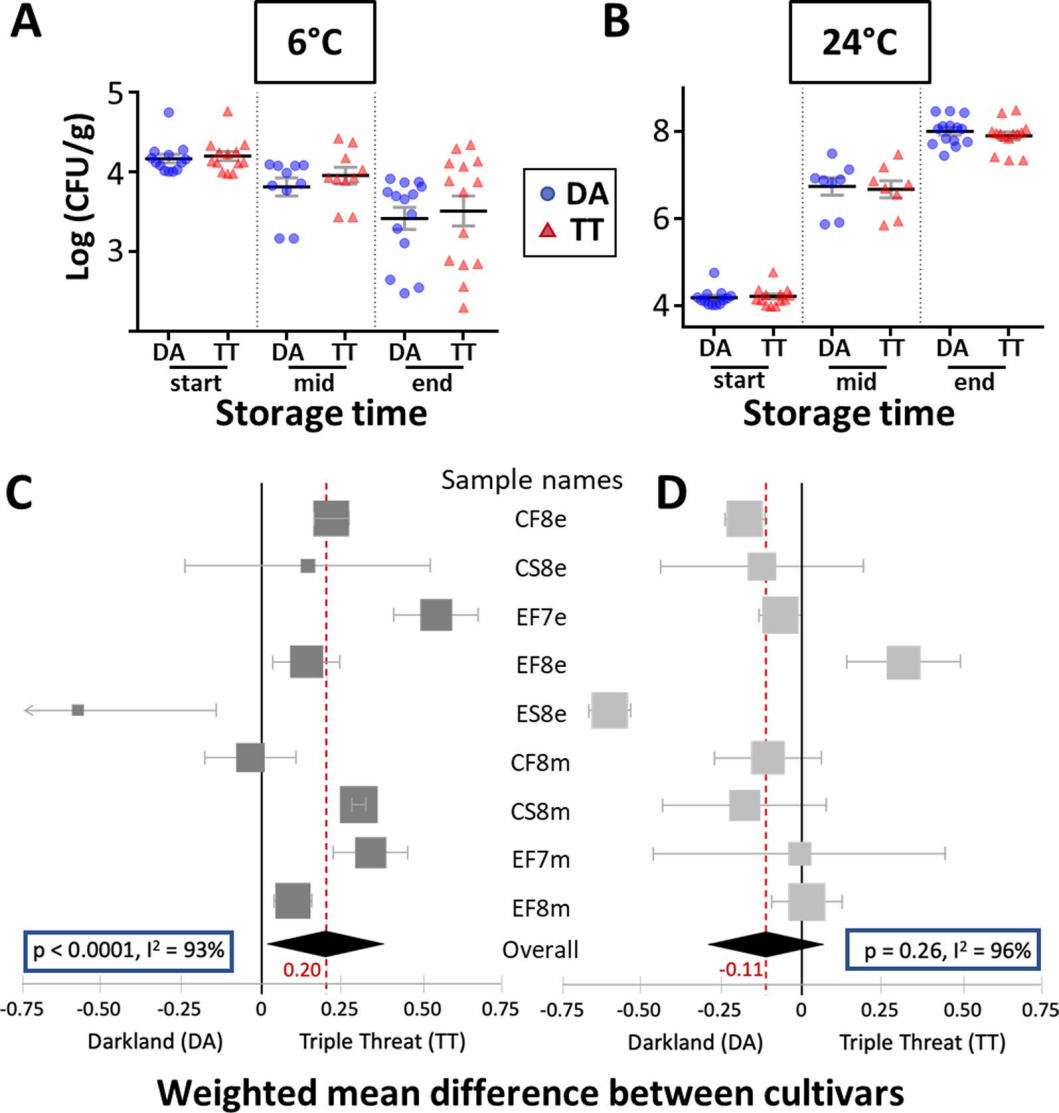
EcO157 population dynamics on postharvest lettuce stored at 6 °C (left panel) and 24 °C (right panel). **a** and **b** Population sizes as Log10-transformed CFU/g cut lettuce for each replicate sample of cultivar DA and TT (226-g MAP bag) immediately after inoculation, and at mid- and endpoint of storage at 6 °C and 24 °C. Black lines and grey bars, means and SEM. **c** and **d** Forest plot of weighted mean differences (WMD) between cultivar TT and DA (TT minus DA) in the change in EcO157 population size [ΔLog10(CFU/g)] from immediately after inoculation (pre-storage) to mid- or endpoint of storage. Overall WMD (black diamonds and red values) based on nine sample types stored at 6 °C (left) and 24 °C (right) across five experiments including different field locations and seasons. Grey rectangles and bars, WMD and CI for each sample type. Sample names: C or E (conventional or experimental field); F or S (fall or spring harvest); 7 or 8 (2017 or 2018 harvest); m or e (mid- or endpoint of storage)

Based on all five experiments, EcO157 population sizes declined on average fivefold over 2 weeks of cold storage (endpoint) (Fig. 5a). At warm temperature, the population sizes increased very rapidly by more than 300-fold within 1 day (midpoint) and over 6000-fold within 2 days (endpoint) (Fig. 5b). Meta-analysis of the difference between DA and TT in the change in EcO157 population size during storage (ΔLog_10_ population sizes) was performed using weighted mean differences (WMDs) and a random effects model. Results revealed that EcO157 population sizes on DA underwent a 1.6 × greater decrease than on TT at 6 °C (*P* < 0.0001) (Fig. 5c), indicating that the human pathogen survived significantly better on the cultivar that had a short shelf life. In contrast, EcO157 population sizes changed similarly on DA and TT at 24 °C (*P* = 0.26) (Fig. 5d), suggesting that lettuce shelf life did not have a significant effect on EcO157 multiplication under conditions of temperature abuse in this study.

### Relation between CO_2_ and EcO157 colonization of MAP lettuce

**Fig. 6:**
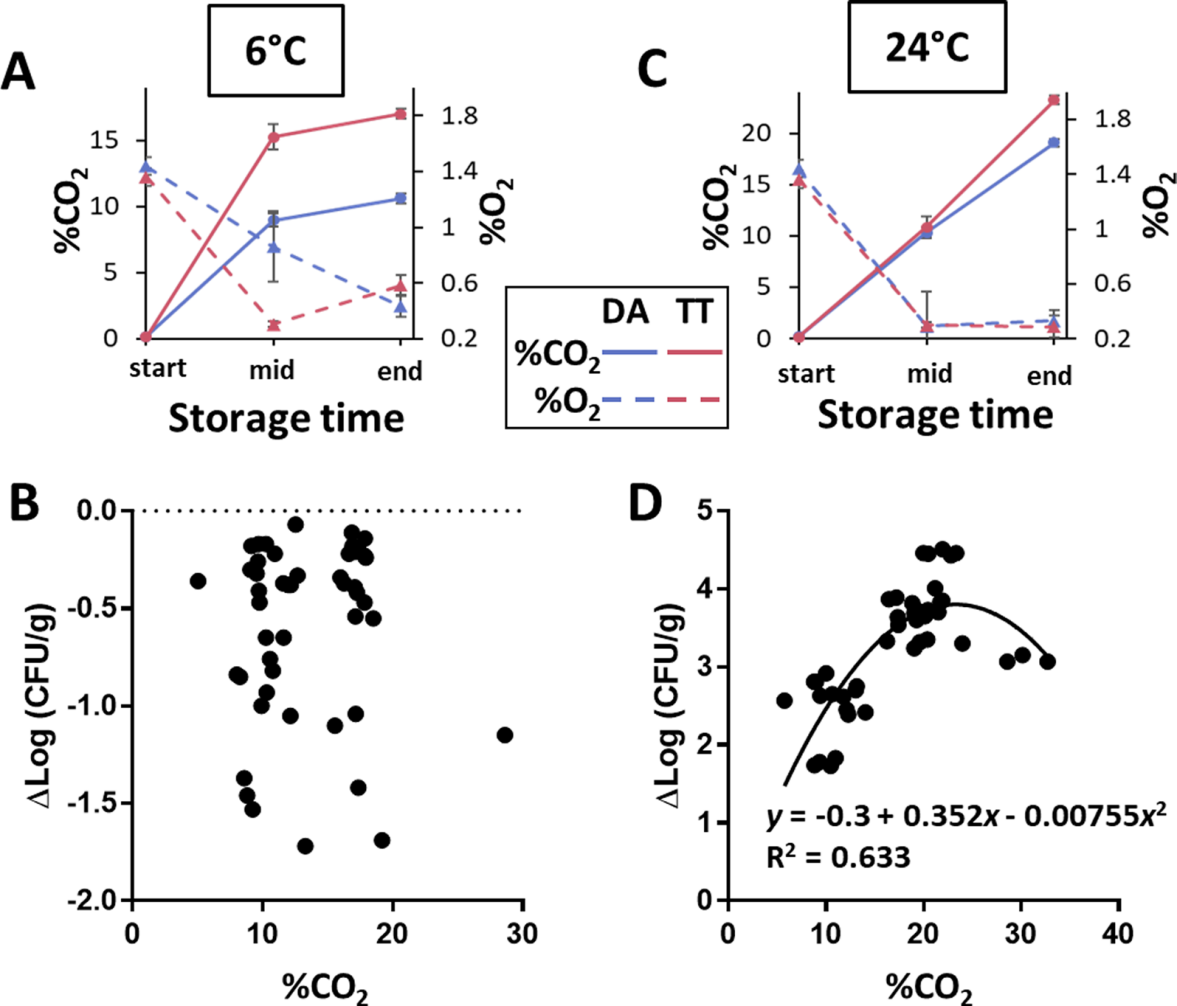
Atmosphere evolution in MAP lettuce bags during storage and effect of CO_2_ content on EcO157 lettuce colonization (left and right panels, 6 °C and 24 °C, respectively). **a** and** c** %CO_2_ and %O_2_ after flushing with N_2_ immediately after inoculation and before sealing the bags, and at mid- and endpoint of sampling. **b** and **d** Scatter plots of the difference between Log10-transformed EcO157 population size at mid- and endpoint of storage and that immediately after inoculation for all samples across five experiments. The line for regression of ΔLog10(CFU/g) over %CO_2_ and descriptive equation, and correlation coefficient are shown for 24 °C samples

The levels of atmospheric O_2_ and CO_2_ were measured in all MAP bags at the start, mid-, and endpoint of storage. At 6 °C, O_2_ was depleted faster in the bags containing TT than DA, with a corresponding faster accumulation of CO_2_ in bags of TT, likely caused by greater tissue deterioration (Fig. 6a). Following 2 weeks of cold storage, the CO_2_ content was 17.0% and 10.7% in TT and DA bags, respectively, and differed significantly (Student’s *t* test, *P* < 0.05). In contrast, O_2_ content was very similar and approximately 0.5% for both cultivars. Despite these differences in CO_2_ evolution and final content in the MAP bags of the two cultivars, no correlation was observed between Logit-transformed %CO_2_ and the change in Log_10_-transformed EcO157 population sizes at mid- and endpoint at 6 °C from that at inoculation time [ΔLog(EcO157 CFU/g)] (Spearman test; *P* = 0.598) (Fig. 6b).

In contrast, atmosphere evolution during MAP storage under warm temperature was highly similar for both cultivars (Fig. 6c). A clear positive relationship between %CO_2_ in the lettuce bags and EcO157 population dynamics was observed, with inhibition however seemingly occurring at higher CO_2_ contents (Fig. 6d). This relationship best fit a 2^nd^ degree polynomial regression, described by y = − 0.3 + 0.352x − 0.00755x^2^ (R^2^ = 0.633), where y is the change in EcO157 population size and x is the content of CO_2_ in percent.

### Recursive partitioning identifies important predictors of EcO157 population dynamics on lettuce

**Fig. 7:**
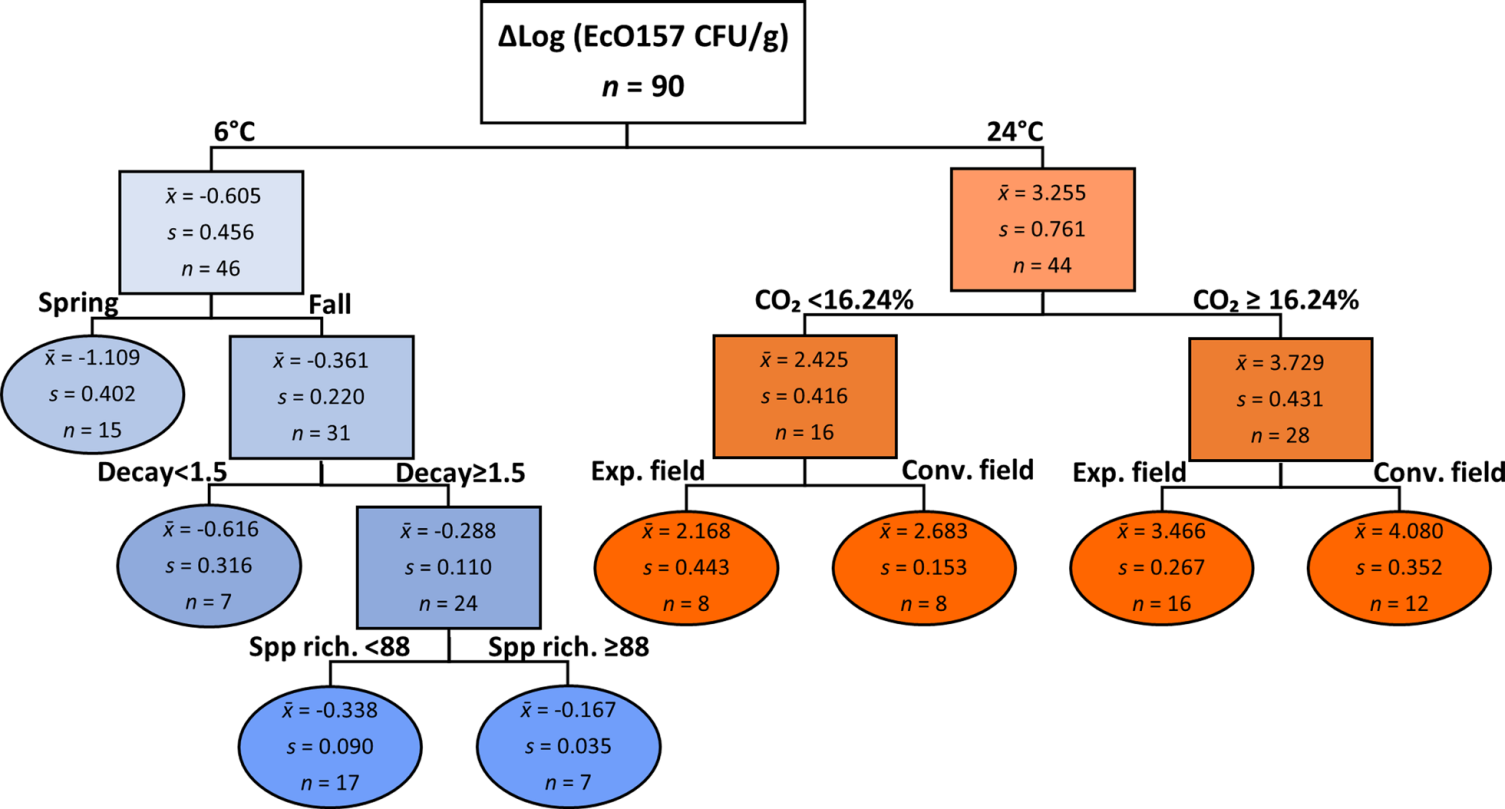
Classification tree model obtained by binary recursive partitioning to predict EcO157 contamination risk of MAP lettuce based on the effect of biotic and abiotic variables on the change in EcO157 population size (ΔLog(CFU/g) during storage at 6 °C and 24 °C. Branching based on cutoff value of Log worth, 2 and minimum n, 7. Spp rich., species richness (derived from microbiome); Exp. and Conv., experimental and conventional field; Decay, rating on scale 1–10

A classification tree was developed using binary recursive partitioning (RP) to determine which independent variables were important predictors of change in EcO157 population size [ΔLog(EcO157 CFU/g)] in MAP lettuce. All input variables are provided in the methods section under description of statistical approaches. The classification model, based on 90 samples from mid- and endpoint of storage, split the tree at first by storage temperature (Fig. 7), an expected result in line with our observations regarding EcO157 population sizes as illustrated in Fig. 5. CO_2_ content in the MAP bags during storage split the 24 °C node, with the greatest multiplication on lettuce occurring at CO_2_ ≥ 16.2%. Under both %CO_2_ branches, the tree was split to final nodes by field type, with greater EcO157 multiplication occurring in the field with conventional agricultural practices than in the experimental field.

**Fig. 8:**
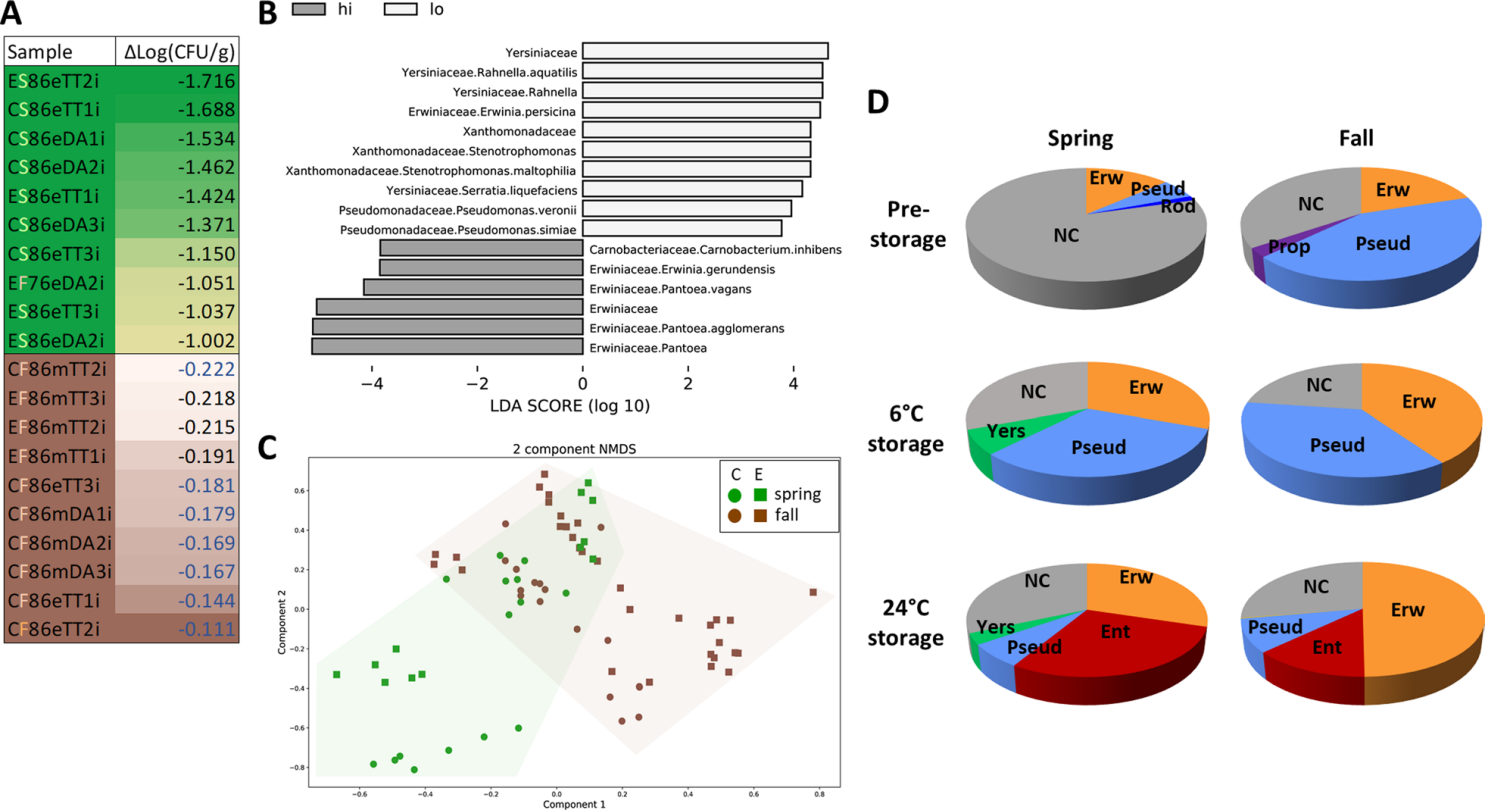
Seasonality of EcO157 survival and core microbiomes on lettuce stored in MAP. **a** Heatmap table of the difference in EcO157 population size [ΔLog(CFU/g)] between either mid- or endpoint of storage at 6 °C and immediately after inoculation. The ten values representing lowest survival (green; nearly all of which, spring samples) and highest survival (brown; all of which, fall samples) out of all inoculated samples across the five experiments (n = 49) are listed. Values in blue font indicate those for samples most significantly associated with highest EcO157 survival also by RP analysis (Fig. 7). **b** Histogram of LDA scores computed using LEfSe analysis for taxa that discriminate between lettuce microbiomes supporting higher and lower survival of EcO157 during cold storage (biomarkers with LDA value ≥ 2.0 depicted). **c** NMDS plot illustrating spring and fall bacterial community structure on lettuce from conventional (C) and experimental (E) fields across the five harvests stored at 6 °C. The metagenomes associated with fall and spring harvests, including inoculated and noninoculated samples, were statistically different (PERMANOVA, *P* = 0.0001). **d** Progression of core and noncore (NC) microbiomes on spring and fall lettuce from pre-storage to end of storage at 6 °C and 24 °C. For 6 °C storage, relative abundance (RA) of each taxa was averaged over mid- and endpoint samples. RA shown for *Erwiniaceae* (Erw), *Pseudomonadaceae* (Pseud); *Enterobacteriaceae* (Ent); *Yersiniaceae* (Yers); *Propionibacteriaceae* (Prop, *Propionibacterium*); and *Rhodocyclaceae* (Rhod, *Dechlorosoma suillum*). In the fall 24 °C-stored lettuce microbiome, *Exiguobacterium sibiricum* (Bacillales) was present at RA = 0.17% and is not depicted. Taxa RA was computed with EcO157 removed for determination of all core microbiomes

On the 6 °C branch of the tree, RP detected season as the most important factor explaining the magnitude of decline in EcO157 population size on MAP lettuce, with fall harvest lettuce predicting the greatest EcO157 survival (mean decline, 2.3-fold) (Fig. 7). Spring harvest-lettuce was a terminal node in the 6 °C branch and was associated with the lowest risk of EcO157 survival (mean decline, 12.9-fold). In the fall season-branch of the decision tree, lettuce deterioration was the most significant determinant of EcO157 survival, revealing that EcO157 survived significantly better at ≥ 15% deterioration (rating ≥ 1.5) on fall-harvested lettuce. At this higher rating, the tree was split to final nodes, showing that fall-harvested lettuce that was deteriorated supported greater survival of EcO157 in a bacterial community with species richness ≥ 88. Remarkably, this group of seven samples posing the greatest risk of EcO157 survival in MAP under cold-storage was among the top ten samples harboring the highest surviving EcO157 populations among all inoculated cold-stored samples in our study (Fig. 8a), supporting RP analysis results. The input variable ‘cultivar’ was pruned from the tree during classification based on the commonly used LogWorth of 2.0 since its effect had a LogWorth of 1.75 (*P* = 0.02). This cut off value is thus lower than the one used in the WMD analysis, which showed a significant difference (*P* < 0.05) between cultivars in EcO157 decline at 6 °C.

### EcO157 survival during 6 °C storage in relation to season, lettuce deterioration, and decay species

The decline in EcO157 population size on cold-stored lettuce ranged from 1.3- to 52.0-fold across all five experiments. Ranking of all inoculated samples stored at 6 °C based on the change in EcO157 population size revealed that the greatest decline occurred in spring-harvested lettuce (with the exception of one fall sample), and the greatest survival was in fall-harvested lettuce (Fig. 8a). The 46 samples that ranked in the middle of the heatmap (data not shown) did not display any seasonality pattern in EcO157 survival. These observations support season as the most significant splitting factor in the 6 °C branch of the decision tree in the model shown in Fig. 7. Additionally, greater EcO157 survival correlated with cold-stored lettuce microbiomes harvested in the fall (Fig. 4a).

Since lettuce deterioration was a significant determinant in EcO157 survival in fall-harvested lettuce (Fig. 7), we investigated relationships between EcO157 survival, deterioration rating, species richness, and RA of major decay species (Additional file 5: Table S3). While lettuce had a deterioration rating ≥ 2 in all ten samples with highest EcO157 survival, all of which were harvested in the fall, rating values varied broadly from 2 to 9. Moreover, only three of these samples (all DA) harbored the well-known soft rot pathogens *P. marginalis and P. viridiflava*, but at a low RA of ≤ 2%. Although certain TT lettuce samples in this group were heavily deteriorated, none had detectable levels of these post-harvest plant pathogens or other decay species, with the exception of one sample with 4% RA of *L. mesenteroides*. Overall, a high deterioration rating in either DA or TT did not de facto imply the presence of decay species, consistent with short shelf life of lettuce being predominantly the result of physiological processes.

Although EcO157 population dynamics were not correlated with the presence of plant pathogens, other bacterial taxa were identified that distinguished microbiomes supporting higher or lower EcO157 survival. *P. agglomerans* and *P. vagans,* along with *Erwinia gerundensis* were significantly more abundant in bacterial communities more favorable to EcO157 survival, while elevated levels of *Rahnella aquatilis* and *Erwinia persicina* were the strongest indicator taxa of microbiomes associated with lowest EcO157 survival (Fig. 8b).

### The lettuce microbiome during 6 °C storage differs by harvest season

The bacterial communities on cold-stored lettuce harvested in the fall and spring differed significantly (PERMANOVA, *P* = 0.0001) (Fig. 8c). In fact, season impacted both the pre-storage core microbiome and shifts in the core microbiome during storage (Fig. 8d, and Additional file 6: Table S4 for species level data). Prior to storage, the spring bacterial communities were very diverse, with noncore taxa contributing an average of 79% RA compared to only 35% RA in fall microbiomes. *Pseudomonadaceae* decreased slightly from 43 to 37% in the fall core microbiome during cold storage but greatly expanded from 6 to 32% RA in the spring core microbiome. With storage, the RA of *Erwiniaceae* increased on lettuce harvested in both seasons and was higher in the fall core microbiome (40%) compared to spring (31%). There was no significant difference in species richness or Shannon index between fall and spring pre-storage bacterial communities, however both alpha indices decreased by midpoint in fall lettuce microbiomes but remained unchanged throughout storage in spring microbiomes (Additional file 7: Fig. S3).

**Fig. 9:**
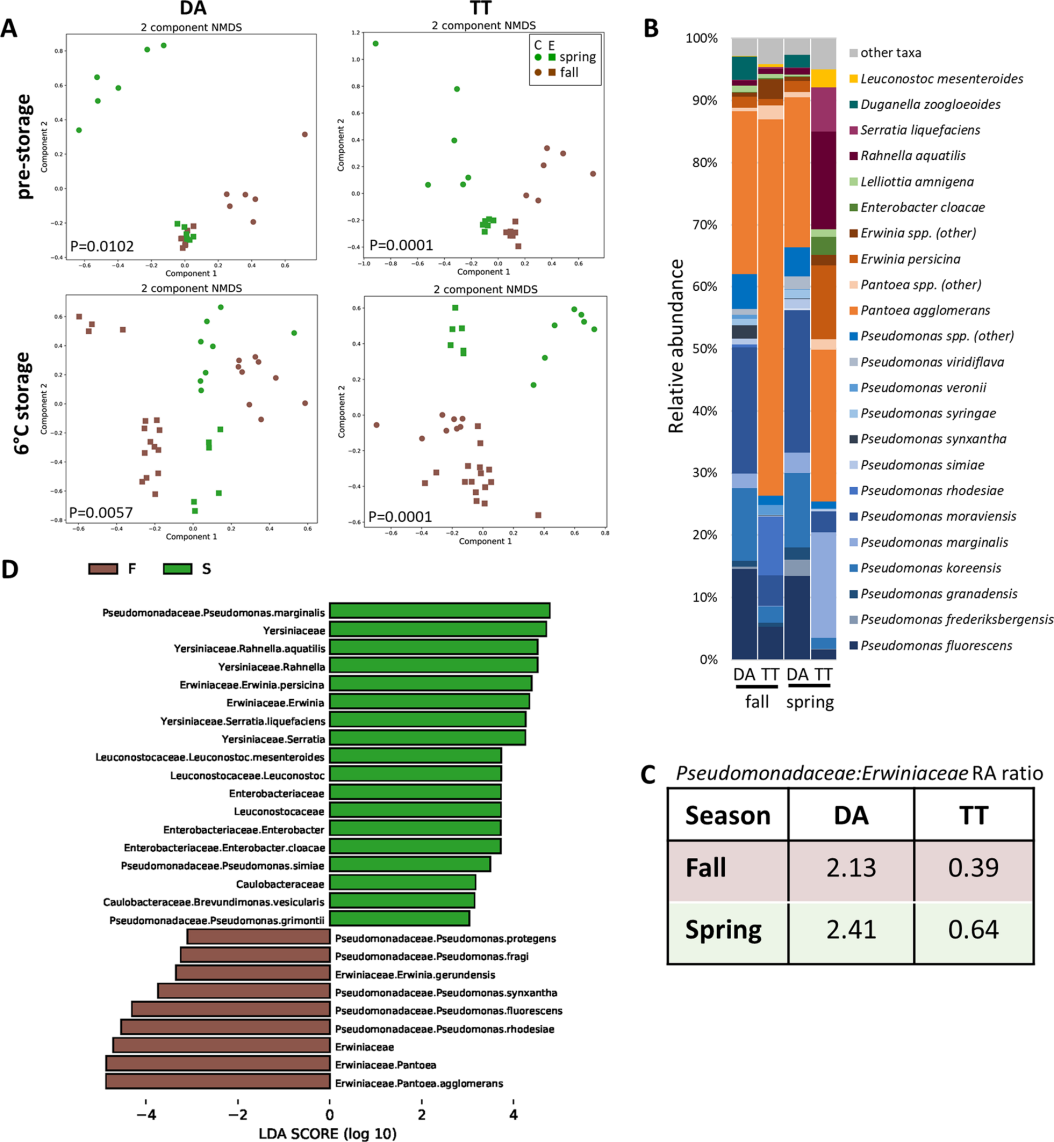
Comparison of seasonal microbiomes on lettuce cultivars stored in MAP. **a** NMDS plots of bacterial community composition by cultivar on processed lettuce pre-storage and with storage at 6 °C (mid- and endpoint included). *P* values for differences in the spring (green) and fall (brown) metagenomes were computed using PERMANOVA and included in the plots. Samples are further categorized as from conventional (C, circles) and experimental (E, squares) fields. **b** Average relative abundance (RA) of bacterial species in lettuce metagenomes by cultivar and season with storage at 6 °C. Species with RA ≥ 0.1 for at least one cultivar and season combination are listed and identified taxa with lower RA are included in ‘other taxa’. **c** Table listing the ratio of relative abundance of *Pseudomonadaceae* to *Erwiniaceae* in the average metagenomes by lettuce cultivar and season with storage at 6 °C. **d** Histogram of LDA scores computed using LEfSe analysis for taxa that are differentially abundant in spring (green) and fall (brown) microbiomes with storage at 6 °C (biomarkers with LDA value ≥ 3.0 depicted). *P. simiae* was found to be a poor biomarker upon closer examination of the RA results

Close examination of the seasonal differences in lettuce bacterial communities revealed that although TT samples contributed to this difference to a greater extent when the cultivars were considered separately (PERMANOVA, *P* = 0.0001), this seasonal trend was also observed for the cultivar DA alone (PERMANOVA, *P* = 0.0057). In fact, these seasonal microbiome differences existed before storage for both cultivars (Fig. 9a). To further elucidate differences in seasonal microbiome composition by cultivar, average bacterial communities were determined (Fig. 9b, Additional file 8: Table S5). The results revealed a much greater RA of *P. agglomerans* in TT in the fall (61%) compared to spring (24%), and greater RAs of *E. persicina, E. cloacae, R. aquatilis, Serratia liquefaciens,* and *L. mesenteroides* in TT in the spring. Albeit more subtle, seasonal contrasts in bacterial community composition were also observed for DA, for example, lower RAs of *Duganella zoogloeoides* and *Lelliottia amnigena* in the spring compared to fall (Fig. 9b). Seasonal differences in the RAs of the various *Pseudomonas* species were also observed for both cultivars (Fig. 9b and Additional file 8: Table S5). At the family level, while more pronounced for TT than DA, the RA ratio of *Pseudomonadaceae* to *Erwiniaceae* was greater in spring than in fall lettuce microbiomes (Fig. 9c).

### Comparison of microbial biomarkers for season and EcO157 population change on lettuce stored at 6 °C

To elucidate season-specific indicator taxa potentially contributing to EcO157 population dynamics, microbial biomarkers differentiating the cold stored fall and spring lettuce microbiomes were determined (Fig. 9d). Several *Pseudomonas* species were identified as enriched in the seasonal bacterial communities. *P. marginalis* was significantly more abundant in spring lettuce microbiomes, whereas *P. fluorescens* was more abundant in the fall. Also, *P. rhodesiae* and *P. synxantha* were exclusively associated with bacterial communities on lettuce harvested in the fall. Importantly, comparison of LEfSe results for distinguishing microbiomes supporting high and low EcO157 survival with those obtained in the seasonal comparison (Figs. 8b and 9d) revealed overlap. In particular, elevated levels of *P. agglomerans* and *E. gerundensis* were associated with both fall bacterial communities and greater EcO157 survival, while microbiomes enriched for *R. aquatilis, E. persicina*, and *S. liquefaciens* were associated with both spring and low EcO157 survival. It is noteworthy that *E. gerundensis* is part of the fall, but not spring, core microbiome, whereas *R. aquatilis* and *E. persicina* are members of the spring, but not fall, core microbiome (Additional file 6: Table S4).

**Fig. 10:**
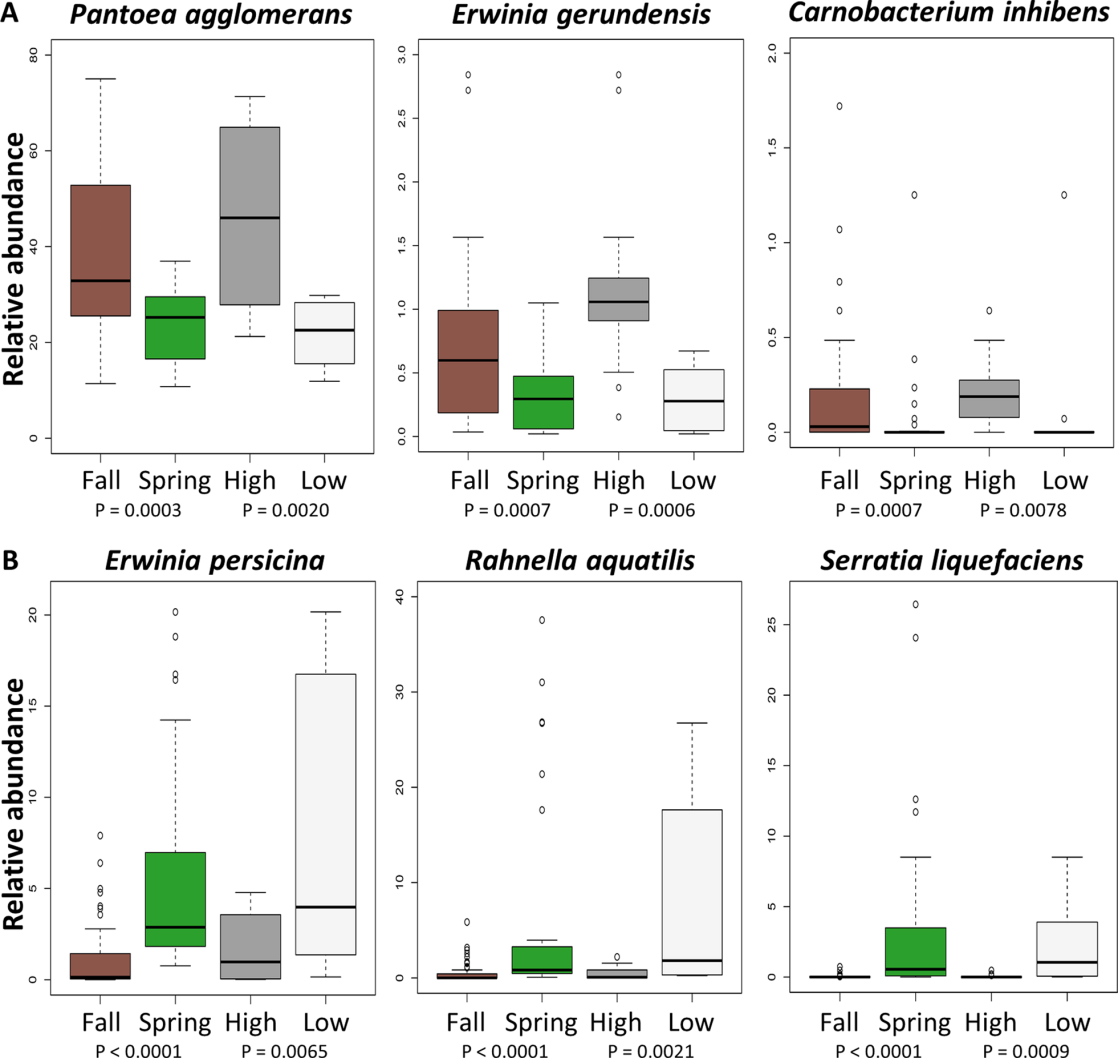
Correlations between relative abundances of biomarker species differentiating harvest season and EcO157 survival in microbiomes on MAP lettuce stored at 6 °C. For high and low EcO157 survival, the 13 samples with greatest EcO157 survival and 10 samples with lowest EcO157 survival were included, and all 6 °C-stored lettuce microbiomes were included in the seasonal comparisons. **a** Indicator species for both fall microbiomes and microbiomes supporting greater EcO157 survival. **b** Indicator species for both spring microbiomes and microbiomes supporting lower EcO157 survival. The bottom and top edges of the boxes mark the 25th and 75th percentiles, and the horizontal line denotes the median. Letters indicate significant differences (Wilcoxon rank sum test with Benjamini–Hochberg adjustment, *P* < 0.05). Pairwise *P* values are given below individual plots

Utilization of LEfSe on the DA and TT cultivars separately revealed that all of the biomarkers in common for season and EcO157 survival were cultivar-specific, with the exception of *E. persicina* (Additional file 9: Figure S4). In addition, the cultivar-specific analysis resulted in the identification of *Carnobacterium inhibens* as a biomarker for fall microbiomes on DA. *C. inhibens* was also found to be in significantly higher RA in bacterial communities associated with greater EcO157 survival (Fig. 8b and Additional file 9: Fig. S4). To further investigate the six identified bacterial species correlated with both season and EcO157 population dynamics, RAs were compared (Fig. 10). The results demonstrate that RAs above approximately 30% and 0.5% for *P. agglomerans* and *E. gerundensis*, respectively, along with presence of *C. inhibens* are all indicative of cold-stored MAP lettuce microbiomes that are more supportive of EcO157 survival (Fig. 10a). Conversely, RAs above approximately 3% and 1% for *E. persicina* and *R. aquatilis*, respectively, along with presence of *S. liquefaciens* are indicative of microbiomes less supportive of EcO157 survival (Fig. 10b). It is important to note that an individual lettuce microbiome that is more supportive of EcO157 survival would not necessarily possess RAs of all three biomarkers above those values because of the cultivar-specificity.

### Seasonality in lettuce stored at 24 °C

Seasonality was also observed in the microbiomes of MAP lettuce stored under warm temperature (PERMANOVA, *P* = 0.0002) (Additional file 10: Fig. S5a). While the relative abundance of *Erwiniaceae* on 24 °C-stored lettuce was similar to that observed at 6 °C in the spring (30%), it represented a considerably greater proportion of the core microbiome in the fall (50%) (Fig. 8d and Additional file 6: Table S4). The *Enterobacteriaceae* also expanded to become a substantial component of the core microbiome under temperature abuse, with over twice the proportional abundance in the spring in comparison to the fall (29% and 13%, respectively). Unlike during storage at 6 °C, the *Pseudomonadaceae* were repressed in relative abundance in both spring and fall at 24 °C. LEfSe identified *Kosakonia cowanii* as a strong biomarker for fall lettuce under warm storage, along with *P. agglomerans*, which was also identified as enriched in cold stored fall lettuce (Additional file 10: Fig. S5b and Fig. 9d). Additionally, as in lettuce stored at 6 °C, *R. aquatilis* and *S. liquefaciens* were enriched in spring microbiomes. Storage under temperature abuse resulted in a decrease in both the Shannon index and species richness in spring microbiomes, while only the Shannon index decreased significantly in the fall microbiomes (Additional file 7: Fig. S3).

### Field effect on lettuce microbiome

Pre-storage bacterial communities clustered separately by field type for each cultivar individually, with a greater variation in beta diversity observed on lettuce that was cultivated with conventional agricultural practices (Fig. 9a). For storage at both 6 °C (Figs. 8c and 9a) and 24 °C (Additional file 10: Fig. S5a), comparisons based on field type for the microbiomes of the two cultivars combined revealed significant differences (PERMANOVA, *P* = 0.0001). It is noteworthy that seven of the ten samples exhibiting highest EcO157 survival at 6 °C were of lettuce harvested from the conventional field (Fig. 8a). However, no apparent field-associated pattern was observed among samples with the greatest EcO157 decline, and field type was not identified as a significant factor in EcO157 survival in the 6 °C branch of the RP classification tree (Fig. 7). In contrast, for lettuce stored at 24 °C, EcO157 population change was associated with field type and significantly greater multiplication occurred on lettuce harvested from the conventional field for both seasons (Figs. 4b and 7). LEfSE analysis identified biomarker species that were enriched in the lettuce harvested from the conventional field. *Leclercia adecarboxylata, L. mesenteroides, Lelliottia amnigena, Enterococcus, and E. persicina* were determined to be significantly enriched in 24 °C-stored lettuce (Additional file 10: Fig. S5c), while *L. amnigena* and various *Pseudomonas* species were enriched in 6 °C-stored lettuce (data not shown).

## Discussion

Leafy greens, in particular lettuce, are the second most common vehicle of STEC infections worldwide despite much effort in minimizing the risk of contamination during production [43, 44]. Lettuce is a highly perishable food product and hence, even minimal processing worsens the stability of the leaf tissue. Although modified atmosphere packaging (1–4% O_2_, 96–99% N_2_) reduces the respiration rate of cut leaf tissue and of its microbiota, lettuce cultivars vary in shelf life and certain genotypes show rapid deterioration evidenced by water-logging and liquefaction of the tissue [40, 45]. In this work, we characterized the microbiomes of two romaine lettuce cultivars with different shelf life harvested in the spring and fall from conventional and experimental fields in Salinas, California. Therefore, our study provides a new window into the changes in the bacterial community and EcO157 colonization of minimally processed lettuce under the influence of several important parameters.

**Role of lettuce shelf life in post-harvest microbiomes and EcO157 colonization**. The lettuce microbiome structure changed significantly during storage at both cold and warm temperatures and differed by cultivar at 6 °C but not at 24 °C. The alteration of the microbial community at 6 °C was not caused by inoculation with EcO157 since the RA of the pathogen in most inoculated samples of both cultivars throughout storage was well below the detection level.

Microbiome alpha diversity, as reflected by the Shannon index, decreased to a greater extent in TT- than DA microbiomes at 6 °C. This indicated that in addition to cold storage in MAP, leaf deterioration and consequently also higher %CO_2_ in the package, select for a less diverse and more uneven microbiome with a greater dominance of species that are better adapted to the physicochemical conditions in degraded plant tissue. Our observations fit the concept of dysbiosis under which microbiomes progress toward less complexity in dysfunctional hosts, including plants [46]. Contrary to in TT, beta diversity in the bacterial community of cultivar DA was stable after midpoint of storage and the microbiome contained a greater predominance of *Pseudomonas* and *Pantoea* than prior to storage. These two genera are highly successful bacterial colonists of the phyllosphere and also dominant members of lettuce leaf microbiomes in the field [5, 9]. The large proportion of *Pseudomonas*, a common psychrophile, has been observed in the bacterial community of cold-stored leafy vegetables such as baby spinach and chard, arugula (rocket), and mixed salads [47–53]. The common plant-associated *Pantoea* spp., *Erwinia* spp., and other species closely related phylogenetically to *E. coli* have been reclassified from the *Enterobacteriaceae* into the new families *Erwiniaceae*, *Pectobacteriaceae,* and *Yersiniaceae* [54], making for a difficult comparative assessment of their RA measured in our study with many previously published phyllosphere microbiomes characterized at the family level.

Importantly, we observed a greater upshift in RA of *Pantoea* spp., a facultative anaerobe, in the rapidly deteriorating cultivar TT than in DA during cold storage in MAP, with a large concomitant decrease in the proportion of *Pseudomonas* species in TT microbiomes. In comparison with DA, the rapid deterioration of TT lettuce caused both faster depletion of O_2_, and more rapid and greater accumulation of CO_2_ in the package, which may have inhibited Pseudomonads in TT samples given their known sensitivity to high CO_2_ levels [55]. These differences in the evolution of bag atmosphere during storage may also explain why MAP-stored TT lettuce harbored an increased relative abundance of *Serratia* spp. and *L. mesenteroides*, two species capable of anaerobic metabolism and associated with vegetable spoilage or fermentation [56, 57].

The soft rot pathogens *P. marginalis* and *P. viridiflava* were present at a low RA (< 2%) in cold-stored fall samples with low levels of decay and high survival of EcO157. In contrast, they were present at a high RA (20–55%) in cold-stored spring samples with high levels of decay and a high decline of EcO157. Also noteworthy are the numerous samples of DA and TT with high levels of deterioration in the absence of any detectable bacterial species known for their plant maceration ability; this included samples with highest and lowest EcO157 survival during 6 °C storage. Soft rot disease was reported to promote multiplication of EcO157 and *S. enterica* in lettuce and cilantro leaves at room temperature in non-MAP conditions [35, 37]. In contrast, our results suggest that the presence or absence of soft rot pathogens in MAP lettuce at 6 °C is not by itself indicative of the level of EcO157 densities, whereas deterioration is a significant factor. Despite the possibility that plant fungal pathogens, which we did not characterize herein, were the cause of the observed degradation in the absence of plant bacterial decay species, previous studies indicate that differences in the shelf life of lettuce cultivars are likely rooted in plant physiological processes and not in microbial degradation [40].

Although the effect of plant tissue deterioration on microbial community succession in produce has been nearly unexplored, the enrichment in *Pantoea*, *Enterobacter* and undetermined members of the *Enterobacteriaceae* in the microbiome of diseased lettuce plants and potato tubers has been reported [7, 58] and O_2_ depletion in macerated leaves at the scale of bacterial cells has been inferred from transcriptome studies [37, 59]. The clear shift in the present study toward greater representation of facultative anaerobes in degrading plant tissue is further evidenced by LEfSe identification of members of the *Erwiniaceae*, *Yersiniaceae*, and *Enterobacteriaceae* as significant microbial biomarkers on both cold-stored and temperature-abused TT lettuce. This suggests common microbial biomarkers in post-harvest conditions under which EcO157, also a facultative anaerobe, is capable of high survival or multiplication on MAP lettuce. A microbial risk assessment study by Söderqvist et al. demonstrated the greater probability of STEC infection from post-harvest processed product, particularly at the end of shelf life [60]. Based on our observations, one may expect a higher level of risk reflected by the presence of a microbial community dominated by bacterial species closely related to *E. coli* such as those belonging to the *Erwiniaceae* (*Pantoea*), *Yersiniaceae* (*Serratia*) and *Enterobacteriaceae*. This would provide an opportunity of developing risk assessment metrics based on an assemblage of biomarker species at the higher level of bacterial community that overcome the pitfalls of using a single *Enterobacteriaceae* or coliform species as indicators of the microbial safety of produce [61]. Such a new approach may prove to be valuable as metagenomics is increasingly being developed and utilized as a tool in microbial food safety [62–65].

Our envfit analysis revealed that deterioration rating, CO_2_ content, and change in EcO157 population sizes were significant co-variates of microbiome structure on cold-stored lettuce. However, no significant correlation was observed between the magnitude of EcO157 population decline and the CO_2_ levels (nor O_2_ levels; data not shown) measured in the MAP bags at mid- and endpoint of storage. This corroborates previous findings that EcO157 survival in romaine and iceberg lettuce was not affected by various atmospheres evolving from cold storage in packaging films of different gas permeability [66].

**EcO157 multiplication, effect on microbiomes, and role of CO2 under temperature abuse**. Temperature abuse, which may occur frequently along the distribution chain, presents one of the greatest risks in food safety [41]. MAP lettuce supported fast multiplication of EcO157 at 24 °C independently of cultivar, likely due to leaf tissue degradation in both cultivars at warm temperature, albeit at lower rates in DA than TT. However, change in EcO157 population size correlated significantly with %CO_2_ in the package at the time of sampling, suggesting that greater CO_2_ content directly or indirectly benefits EcO157 on MAP lettuce under temperature abuse. The quadratic relationship between these two variables, as detailed in the Results, suggests that the EcO157 population may multiply tenfold on cut lettuce at 24 °C in an atmosphere evolving from 0% CO_2_ at the time of packaging to 4.1% CO_2_. Given the low infectious dose of EcO157, with an estimated range of 1–100 cells [67], MAP lettuce kept at room temperature may be associated with a greater risk should the product be contaminated with even very few pathogen cells.

The core microbiome of MAP lettuce during storage at 24 °C (with EcO157 removed from the microbiomes) was enriched proportionally and with a greater diversity in members of the *Erwiniaceae* and *Enterobacteriaceae*. This is in agreement with our observation that EcO157, also a facultative anaerobe, thrived under these rapidly CO_2_-accumulating and O_2_-depleting conditions. In contrast, the *Pseudomonadaceae* decreased in their overall representation in the core microbiome under these conditions. The role of CO_2_ as a major driver of microbial behavior in MAP lettuce under temperature abuse was further supported by results of our envfit and recursive partitioning analyses.

While the starting EcO157 inoculum level was much higher in our study than the level of EcO157 contamination one would expect on processed lettuce, and temperature abuse of packaged lettuce at 24 °C may rarely take place for very prolonged periods of time, microbial dynamics of a similar nature may occur at smaller spatial and time scales, or as punctuated events, throughout distribution, retail and after purchase by consumers. We previously reported multiplication of EcO157 greater than tenfold within only 4 h upon contamination of shredded lettuce held at 24 °C [35].

**Seasonality in EcO157 survival, microbiomes, and deterioration in lettuce stored at 6 °C**. Recursive partitioning analysis revealed that season was the main driver of EcO157 behavior on cold-stored MAP lettuce as it was the most important splitting factor for change in the pathogen population size in the 6 °C branch of the decision tree, while it was not a factor in EcO157 multiplication at warm temperature. Seasonality was also evident in the lettuce microbiome from post-processing through cold and warm storage. Although composed primarily of *Pseudomonas* spp. and *Pantoea* spp., the fall core microbiome prior to storage was much larger than in the spring. This indicates that the pre-storage spring lettuce microbiome is much more fluid, with a larger number of variable taxa among samples, whereas the fall microbiome appears to have undergone more selective pressure to host primarily two highly successful plant colonists as the majority of the microbiome. An additional notable trend is the relatively stable and high RA of the *Pseudomonadaceae* in the fall lettuce bacterial community throughout cold storage compared with its upward shift in the spring lettuce. Hence, the pseudomonad subpopulation and the broader bacterial community were highly dynamic throughout storage on spring lettuce, which was also the most inhibitory to EcO157 survival. The RA of the *Pseudomonadales* was shown previously to be negatively correlated with viable counts of EcO157 in bagged baby spinach stored at 8 °C [51]. One could hypothesize that beyond endpoint proportionality, the dynamic representation and diversification of the core *Pseudomonas* species in the postharvest spring lettuce, from *fluorescens* prior to storage to include 7 other species during storage, effected a microbial environment that was less hospitable to EcO157 than that in the fall. Seasonal differences in lettuce communities were already present immediately before harvest in our studies (unpublished data). These observations along with weather parameters during the spring and fall harvest are the topic of an upcoming research article. Seasonality in field lettuce microbiomes in California have been reported previously, albeit this difference was evidenced in the proportion of Firmicutes to Proteobacteria [5, 9].

Our identification of microbial biomarkers indicative of bacterial communities in which EcO157 had high and low survival during cold storage, along with the cultivar-specific comparison of fall and spring microbiomes, indicate that different cultivars host separate microbial communities that are more or less hospitable to EcO157. One exception to this was the enrichment of *E. persicina* in spring microbiomes on both DA and TT lettuce as well in microbiomes of samples with lower EcO157 survival. *E. persicina* inhibited the growth of *Salmonella enterica* on alfalfa sprouts [68] and of EcO157 in vitro and on spinach leaves [69]. While EcO157 did not multiply on cold-stored MAP lettuce, it is possible that an inhibitory factor produced by *E. persicina* cells present on spring-harvested lettuce impacted EcO157 survival.

Importantly, our observed seasonality of EcO157 behavior and microbiome composition in MAP lettuce provides the first biological basis for the higher incidence of outbreaks of STEC infection linked to lettuce harvested in the late growth season in coastal California (September–October) [33, 34]. Our results are in agreement with ‘fall season’, ‘processing and storage’, and ‘end-of-shelf life’ presenting the highest risks of EcO157 infection, as identified by quantitative microbial risk assessment based on numbers of *Enterobacteriaceae* and *E. coli* in spinach and rocket (arugula) cultivated in Sweden in 2014–2016 [60]. With regard to “end-of-shelf life” in the latter study, it is also fitting that recursive partitioning herein identified “deterioration (> 15%)” as the most significant factor after “fall harvest” in the survival of EcO157 on cold-stored lettuce. Despite a greater seasonal difference for the cultivar TT compared with DA, both DA and TT microbiome compositions individually displayed significant seasonality prior to and during storage. Furthermore, DA samples harvested in the fall were among the top ten samples supporting the smallest decline in EcO157 densities at 6 °C. DA was also more prone to post-harvest deterioration at 6 °C in the fall than in the spring, a trend observed previously with other lettuce cultivars known to have long shelf life [40]. Processing of leafy greens causes considerable tissue damage [53], which may increase leaf tissue respiration rate and accelerate senescence [70]. Hence, it is tempting to surmise that the seasonality of lettuce-linked STEC infections may be rooted at least partly in seasonal aspects of lettuce physiology that remodel the leaf microbiome and also enhance its suitability as a habitat for this human pathogen.

## Conclusions

Collectively, our findings reveal that season, lettuce shelf life, factors intrinsic to MAP storage, and cultivation practices all impact bacterial communities as well as EcO157 behavior upon its introduction into minimally processed lettuce stored in MAP. We also demonstrate that the lettuce microbiome and EcO157 behavior during storage are correlated. Fall season and lettuce deterioration were associated with greater EcO157 survival during storage at 6 °C and furthermore, modulated the microbiome. Under temperature abuse, EcO157 multiplied rapidly in both cultivars and independently of season whereas the lettuce microbial communities showed seasonality. *Pantoea agglomerans* was particularly enriched in deteriorated lettuce at 6 °C, under temperature abuse, and in the fall microbiomes at both storage temperatures. Large efforts are currently focused on understanding the zoonotic and environmental factors involved in the seasonal occurrence of STEC outbreaks linked to lettuce. In facing this challenge, our results clearly indicate that it will be critical to also decipher the effect of season on the plant host, its residing microbiota, and their interaction with EcO157, in order to improve the microbial safety of lettuce and public health.

## Methods

**Experimental design**. A schematic description of our study and variables is provided in Fig. 1. Briefly, two romaine type lettuce cultivars that differ in shelf life were planted in a commercial lettuce field under conventional cultivation and in an experimental field, in Salinas, CA. The lettuce was grown and harvested in the spring and fall. The lettuce heads were processed as fresh-cut product, inoculated or noninoculated, packaged in MAP, and stored at cold or warm temperature. EcO157 was recovered and population sizes were quantified, and microbiomes were collected from the lettuce immediately prior to storage in MAP and at mid- and endpoint of storage (Fig. 1). Five replicate experiments were conducted in total, that is from five different field trials, as detailed below. Samples in our study were labeled with the following scheme as consecutive letters or digits in the name: C or E (Conventional or Experimental field); F or S (Fall or Spring harvest); 7 or 8 (2017 or 2018); no digit, 6 or 24 (pre-storage, or storage at 6 °C or 24 °C); z, m, e (microbiome collected at T(0) i.e. pre-storage, mid-, or endpoint of storage); DA or TT (cultivar Darkland or Triple Threat); 1, 2 or 3 (replicate sample number consisting of each replicate lettuce bag per condition per sampling time within each experiment); and n or i (noninoculated or inoculated with EcO157). Thus, a sample harvested in the conventional field in fall 2018, stored at 6 °C, analyzed at midpoint of storage, and obtained from cultivar DA, replicate bag 2 that was inoculated with EcO157 would be named: CF86mDA2i.

**Strain and culture conditions**. A spontaneous rifampin-resistant mutant of a naturally Shiga toxin-minus EcO157 strain ATCC 43888 [71] was used in this study. This strain was cultured to early stationary phase at 28 °C in Luria Bertani—half salt (0.5% NaCl) (LBHS) broth from a colony grown on LBHS agar containing rifampin at 100 μg/mL (LArif). For inoculum preparation, the cells were washed twice in potassium phosphate buffer (KPB) (10 mM, pH 7.0), and resuspended in KPB (1 mM, pH 7.0) at 5 × 10^5^ CFU/mL based on OD_600_.

**Plant material**. Lettuce (*Lactuca sativa* L.) romaine-type Triple Threat (TT) and Darkland (DA), two cultivars with short and long shelf life, respectively [72], were planted at three different locations: field plot A at the Spence experimental field, USDA, ARS, Salinas, CA, hereafter referred to as “experimental field”; and as plots embedded in commercial lettuce fields B and C, Salinas, CA, hereafter referred to as “conventional field”. Fields B and C were approximately 1.5 km apart and all three fields were on sandy loam soil and experience highly similar weather conditions. Lettuce was planted as seed on 1-m-wide raised beds in two lines 35-cm apart, and plants were thinned to 30-cm distance one from another within the rows at ca. 4 weeks post-seeding. In the experimental field, plants were overhead irrigated with well water throughout the growth season, herbicides were applied prior to planting, insecticides were applied as needed, but no fungicides were used. Plants grown in commercial fields B and C were overhead irrigated to mid-season, followed by drip irrigation until harvest. Other details of agricultural practices in the commercial fields are proprietary information, but it is expected that herbicides were used, and insecticides and fungicides were applied to the lettuce plants as per standard practice. Harvest dates for spring and fall per location were as follows: (1) field A, November 7, 2017, and June 5 and November 7, 2018; (2) field B, May 30, 2018; and (3) field C, October 30, 2018.

**Plant harvest and processing**. Lettuce plants were harvested in the early morning, at random across the beds and along the rows by cutting the stem with a knife while wearing gloves. Gloves were changed for harvest of different cultivars. Approximately 50 heads of each cultivar were packed in boxes separately and taken immediately to the USDA, ARS Research Station, Salinas, CA. Using gloves, wrapper leaves and unhealthy material were cut away and the heads were sliced with an Easy LettuceKutter (NEMCO, Hicksville, OH) into 25 × 25 mm pieces. The cut lettuce was washed with gentle agitation for 3 min at 5 kg per 50 L water containing 10–15 mg/L free chlorine final, as per measurement with a Lovibond® MD 100 Photometer (Tintometer Inc.) in order to minimize cross-contamination [73]. The lettuce was rinsed for 1 min three consecutive times in fresh municipal water (FC, 0.07 mg/mL; pH 6.7). Material from different cultivars was kept separate and washed and rinsed in different water. The lettuce was centrifuged at 2 ×*g* for 5 min in a food processing centrifuge (FP-35, Bock Engineered Products, Toledo, OH) and immediately stored in large coolers at 4 °C for transportation to the ARS Center, Albany, CA for further use.

**Inoculation of lettuce and storage**. Cut lettuce was placed at 226 g/bag made to size (24 cm × 24 cm) with proprietary MAP polymer film specific for fresh-cut lettuce obtained from a commercial lettuce processor. Two mL of inoculum suspension, or KP (1 mM, pH 7.0) for mock inoculation, was added to the cut lettuce. Inoculation with EcO157 resulted in a population density ranging from 1 to 5 × 10^4^ CFU/g lettuce in the bag, depending on the accuracy of the OD_600_ reading against the standard curve of CFU/mL during inoculum preparation. Following inoculation, the bag was partially sealed, flushed 3 × with N_2_, and then fully sealed. The atmosphere was measured in each bag immediately after sealing and again at sampling time with an O_2_ and CO_2_ Pac Check 650 EC sensor probe (Mocon, Brooklyn Park, MN) and verified to be 1–2% O_2_. Three replicate bags were prepared for each cultivar per sampling time, except in the fall 2017 experiment, in which two replicate bags were used. In total, 50, 78 and 56 samples were prepared for analysis before storage, and under 6 °C- and 24 °C-storage, respectively. Bags were stored in a randomized design in a cold room at 6 ± 2 °C, or in an incubator at 24 ± 1 °C. EcO157 population sizes were measured immediately after inoculation and at times indicated below. Midpoint sampling time of lettuce stored at 6 °C was ca. 7 days, by which time lettuce deterioration was macroscopically detectable in at least one third of the lettuce pieces in cultivar TT (short shelf life); at 24 °C, midpoint sampling was at 14–24 h of storage when TT lettuce showed small but macroscopically discernible deterioration at the margins. Endpoint sampling was ca. 14 days and 38–66 h at 6 °C and 24 °C, respectively, when cultivar TT showed a large amount of deterioration in most bags.

**Shelf life ratings**. Each lettuce bag was visually evaluated at sampling time before processing to assess deterioration on a scale of 0 through 10 that corresponds to the estimated percentage of deteriorated tissue divided by 10 [40].

**EcO157 and microbiome recovery**. Each sample of 226 g lettuce (one bag) was processed for 2 min in 600 ml KPB (10 mM, pH 7) in a stomacher bag with a membrane in a JumboMix 3500 W Lab Blender (Interscience, Woburn, MA). Two aliquots of the suspension were dilution plated onto LArif, plates were incubated at 37 °C and CFU counts performed to quantify EcO157 population sizes. Microbiomes were obtained by successive filtration of 200-mL aliquots of the suspensions recovered from the lettuce as described above. All suspensions and filtrates were kept at 4 °C throughout the procedure. The suspension was filtered through a 80 µM nylon membrane, then through a 11 µM nylon membrane. At least two replicate 20-mL samples of this filtrate were filtered each through a 10 µM Isopore membrane. Replicate filtrates were centrifuged at 10 °C, 20 min, 19,000×*g*. The supernatant was removed and the microbial pellets stored at − 80 °C until DNA extraction.

**Microbiome DNA extraction and sequencing**. DNA was extracted from the microbial pellets using the DNeasy PowerSoil Kit (Qiagen, Germantown, MD) with the modifications of an increased vortex time of 20 min for all samples and an extra wash step. Sequencing libraries were constructed using the Nextera DNA Flex Library Prep Kit (Illumina, San Diego, CA), and were sequenced using 150 bp paired-end sequencing on an Illumina NextSeq platform, generating an average of 50.8 million reads per sample. Sequence reads were quality checked by FastQC 0.11.8 [74] then trimmed for quality and adapter sequences were removed using the NexteraPE adapter file in Trimmomatic 0.38 [75].

### Microbiome and statistical analyses

*Microbiome analyses*. Determination of bacterial composition from shotgun sequencing was conducted using custom C++ programs and a *k*-mer signature database (github.com/mmammel8/kmer_id) [63]. The *k*-mer database used in this work contains 5900 target entries, each consisting of approximately 40,000 (range 44–80,000) unique *k*-mers. The database includes 1100 different bacterial genera, and 3500 species. Confirmation of low-level species calls were made by BLAST analysis of reads. Core microbiomes were determined by averaging the relative abundance (RA) of only the bacterial taxa present above 0.02% in all samples within a group. Average microbiomes include all taxa above 0.02% RA irrespective of whether the taxa are present in all samples within a group. To determine alpha diversity indices, RAs of subspecies identified were added into the corresponding species RA, then to reduce noise, only species with at least 0.02% RA were retained. The count for each species was determined by multiplying the RA by the total number of identified reads. The observed number of species was counted and rarefied in R version 4.0.2 with the vegan library v2.5-6 (https://CRAN.R-project.org/package=vegan). Statistical comparisons of alpha diversity indices were performed using the Wilcoxon rank sum test and adjusted *P* values using the Benjamini–Hochberg method. For determination of beta diversity, the Bray–Curtis dissimilarity matrix for pairwise comparison of each bacterial community was determined in Python 3.7.3 and used as input into the sklearn library manifold function for Non-Metric Multidimensional Scaling (NMDS). Significance of group clustering was determined by the skbio library permutational multivariate analysis of variance (PERMANOVA) function [76] with 9999 iterations. For most samples (38/46), EcO157 was not detected in the inoculated lettuce metagenomes during 6 °C storage and never exceeded 0.072% RA when detected. Therefore, where indicated, EcO157 was removed from the bacterial community for computing RAs. Plotting of environmental and EcO157 population vectors on NMDS plots was performed in R using the envfit function in the vegan library. Identification of bacterial species characterizing differences between groups of lettuce metagenomes was performed by linear discriminant analysis (LDA) Effect Size (LEfSe) [77] using default parameters. To identify bacterial taxa that distinguish microbiomes supporting higher or lower EcO157 survival, LEfSe analysis was performed on subsets of sample microbiomes. The ten lowest survival values formed a separated cluster from the middle values, whereas the 13 highest survival values were a distinct cluster from the middle values, thus those two groups were used for the analysis.

Three samples were noted to be possible outliers based on their distance from replicate and related samples in NMDS plots. That difference was determined to be mainly due to the RA of *Pseudomonas* spp. in the microbiomes. *Pseudomonas marginalis* dominated the microbiomes of CS86eTT2i and CS824eTT3n, while the CF86mTT3i microbiome had an unusually low RA of *Pseudomonas* spp. All three samples were determined to be outliers based on the interquartile rule method and were removed from all analyses.

*EcO157 population size analyses*. Meta-analysis of Δlog CFU data was performed with Review Manager (RevMan) v.5.4 (The Cochrane Collaboration, 2020) using weighted mean differences (WMDs) and a random effects model. More precisely, the change in Log10-EcO157 population size at mid- or endpoint of storage versus that at inoculation time prior to storage was quantified for each TT and DA sample in each experiment. The difference in this change in population size between TT and DA was then computed by WMD analysis. Recursive partitioning (RP) was performed with JMP Pro 15.0.0 (SAS Institute, Carry, NC, USA). Partitioning was stopped at LogWorth of 2.0 (*P* = 0.01) or the minimum size split of 7. The response variable in the analysis, ΔLog (EcO157 CFU/g), was the difference in population size as Log-value of EcO157 CFU/g at mid- and endpoint storage times and at T(0), the sampling time immediately after inoculation (pre-storage sampling). The independent variables were field (conventional production field vs experimental station field), season (spring vs. fall), cultivar (DA vs. TT), storage temperature (24 °C vs. 6 °C), sample bag atmosphere (%O_2_, %CO_2_, and %N_2_), and deterioration rating; the microbiome-derived variables included in the analysis were the alpha diversity measures of Shannon index and species richness, as well as the RA of *Pseudomonadaceae* and *Erwiniaceae*, the two dominant families consistently detected across all samples. EcO157 multiplied to represent a large part of the microbiome during storage at 24 °C and thus, microbiome-derived variables were not included to generate the 24 °C branch of the partitioning tree given that they were not variables that are independent of the response variable ΔLog (EcO157 CFU/g).

## Supplementary Information

**Additional file 1: Table S1.** Table of the relative abundance of bacterial decay species in the microbiome of all lettuce samples (A) before storage, (B) during storage at 6 °C, and (C) during storage at 24 °C.

**Additional file 2: Table S2.** PERMANOVA results on the effect of cultivar, season, field type, and EcO157 inoculation on the lettuce microbiome composition at each sampling time at 6 °C and 24 °C.

**Additional file 3: Fig. S1.** Indicator taxa distinguishing microbiomes associated with romaine lettuce cultivars DA and TT with cold storage. Histogram of LDA scores computed using LEfSe analysis for taxa differentially abundant, including all five harvests, between the metagenomes of cultivars DA and TT after storage at 6°C (biomarkers with LDA value ≥ 3.5 depicted).

**Additional file 4: Fig. S2.** Change in the lettuce microbiome during storage at 24°C in MAP as shown by (A) NMDS plot of TT and DA microbiome before and after storage, (B) relative abundance of bacteria genera on inoculated, inoculated (with EcO157 removed from microbiome analysis), and noninoculated samples, and (C) NMDS beta diversity plot revealing the effect of inoculated EcO157 on lettuce microbiome composition.

**Additional file 5: Table S3.** Table of lettuce samples with lowest and highest EcO157 survival during 6°C storage, in relation to species richness, decay rating, and relative abundance of various decay species.

**Additional file 6: Table S4.** Table of bacteria species forming the core microbiome of fall- vs spring-harvested lettuce, before and during storage at 6 °C and 24 °C.

**Additional file 7: Fig. S3.** Seasonal comparison of lettuce microbiome alpha diversity measures. Observed species richness and Shannon Index of fall and spring microbiomes, with inoculated EcO157 removed for analysis, at pre-storage and at midpoint and endpoint during storage at the indicated temperatures. The bottom and top edges of the boxes mark the 25th and 75th percentiles, and the horizontal line denotes the median. Letters indicate significant differences (Wilcoxon rank sum test with Benjamini-Hochberg adjustment, *P* < 0.05).

**Additional file 8: Table S5.** Average relative abundance of bacteria species in the microbiomes of lettuce cultivars TT and DA by harvest season.

**Additional file 9: Fig. S4.** Comparisons of indicator taxa in bacterial communities on lettuce stored in MAP at 6°C based on seasonal differences or survival of inoculated EcO157. Histograms of LDA scores computed using LEfSe analysis for taxa that are differentially abundant are depicted for lettuce harvested in the fall (red) or spring (green) for the two cultivars individually and for lettuce supporting either higher (dark grey) or lower (light grey) survival of EcO157. The green boxes indicate a common biomarker for both DA and TT spring microbiomes and microbiomes supporting lower EcO157 survival. The red boxes indicate biomarkers common to fall microbiomes of one cultivar and lettuce microbiomes supporting higher EcO157 survival.

**Additional file 10: Fig. S5.** Comparisons by season and field for MAP lettuce stored at 24°C. **a** NMDS plot of endpoint bacterial community structure for lettuce harvested in the spring (green) or fall (brown) and grown in the conventional field (C) or experimental field (E). EcO157 was removed for beta diversity determination of inoculated samples. The endpoint microbiomes differed by both season (PERMANOVA, *P* = 0.0002) and field (PERMANOVA, *P* = 0.0001). **b** Histogram of LDA scores computed using LEfSe analysis for taxa differentially abundant between the endpoint microbiomes on lettuce harvested in the fall and spring (biomarkers with LDA value ≥ 3.5 depicted). **c** LDA scores computed using LEfSe analysis for taxa differentially abundant between the endpoint microbiomes on lettuce harvested in the conventional field and experimental field (biomarkers with LDA value ≥ 3.5 depicted). Inspection of individual relative abundance plots revealed P. agglomerans, P. protegens, K. cowanii, P. marginalis, Enterococcus, and S. liquefaciens to be poor indicator taxa in the field comparison.

## Data Availability

All sequence data generated in this work is available at the NCBI Sequence Read Archive under BioProject number PRJNA716697.
