# Supplementary figures and images for: Seasonality, shelf life and storage atmosphere are main drivers of the microbiome and E. coli O157:H7 colonization of post-harvest lettuce cultivated in a major production area in California
Source: Environ Microbiome. 2021 Dec 20;16:25. doi: 10.1186/s40793-021-00393-y (PMC8686551; doi:10.1186/s40793-021-00393-y)

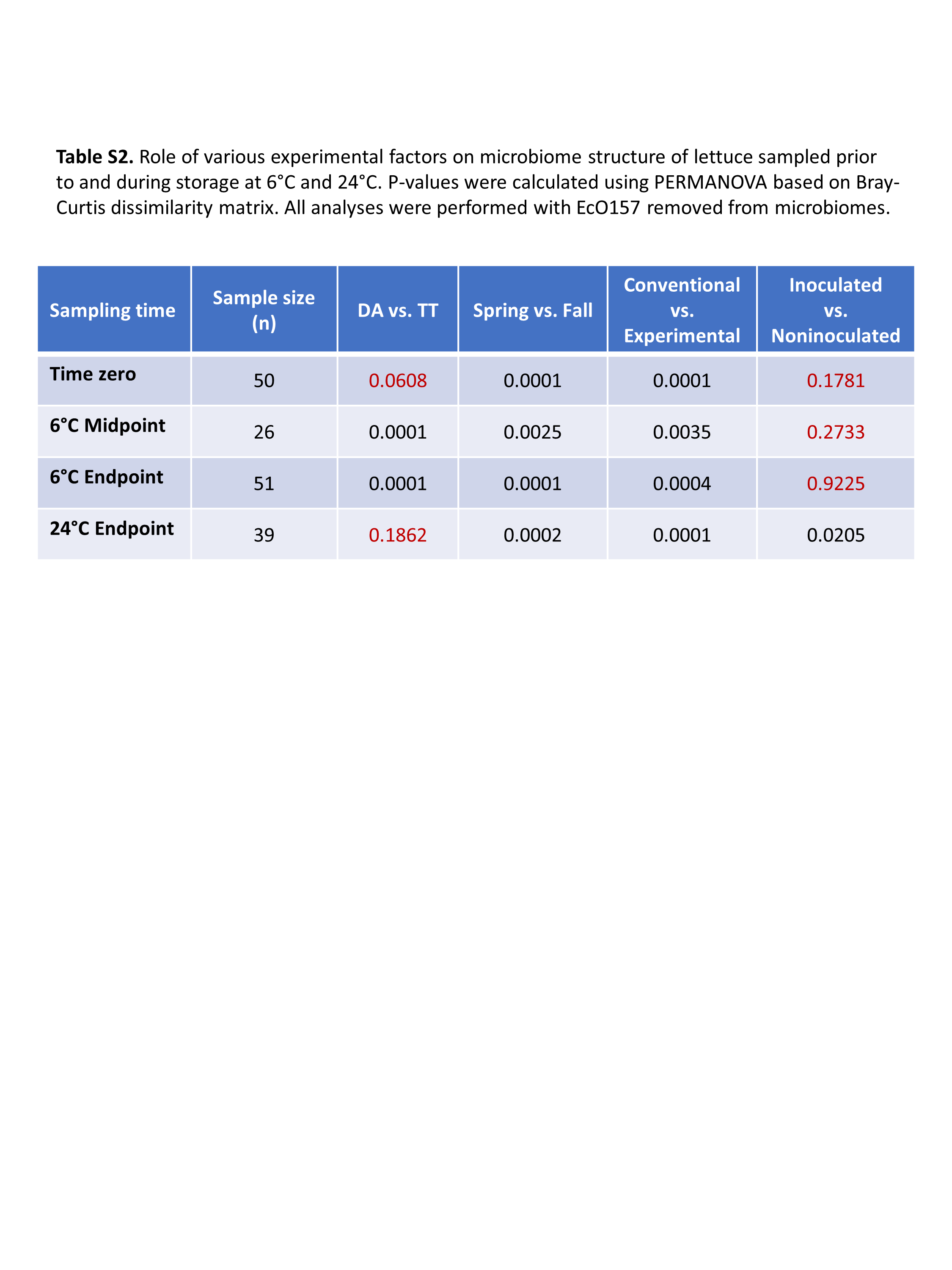

Supplement: Supplementary file 2 — Additional file 2: Table S2. PERMANOVA results on the effect of cultivar, season, field type, and EcO157 inoculation on the lettuce microbiome composition at each sampling time at 6 °C and 24 °C. [file 40793_2021_393_MOESM2_ESM.tif]

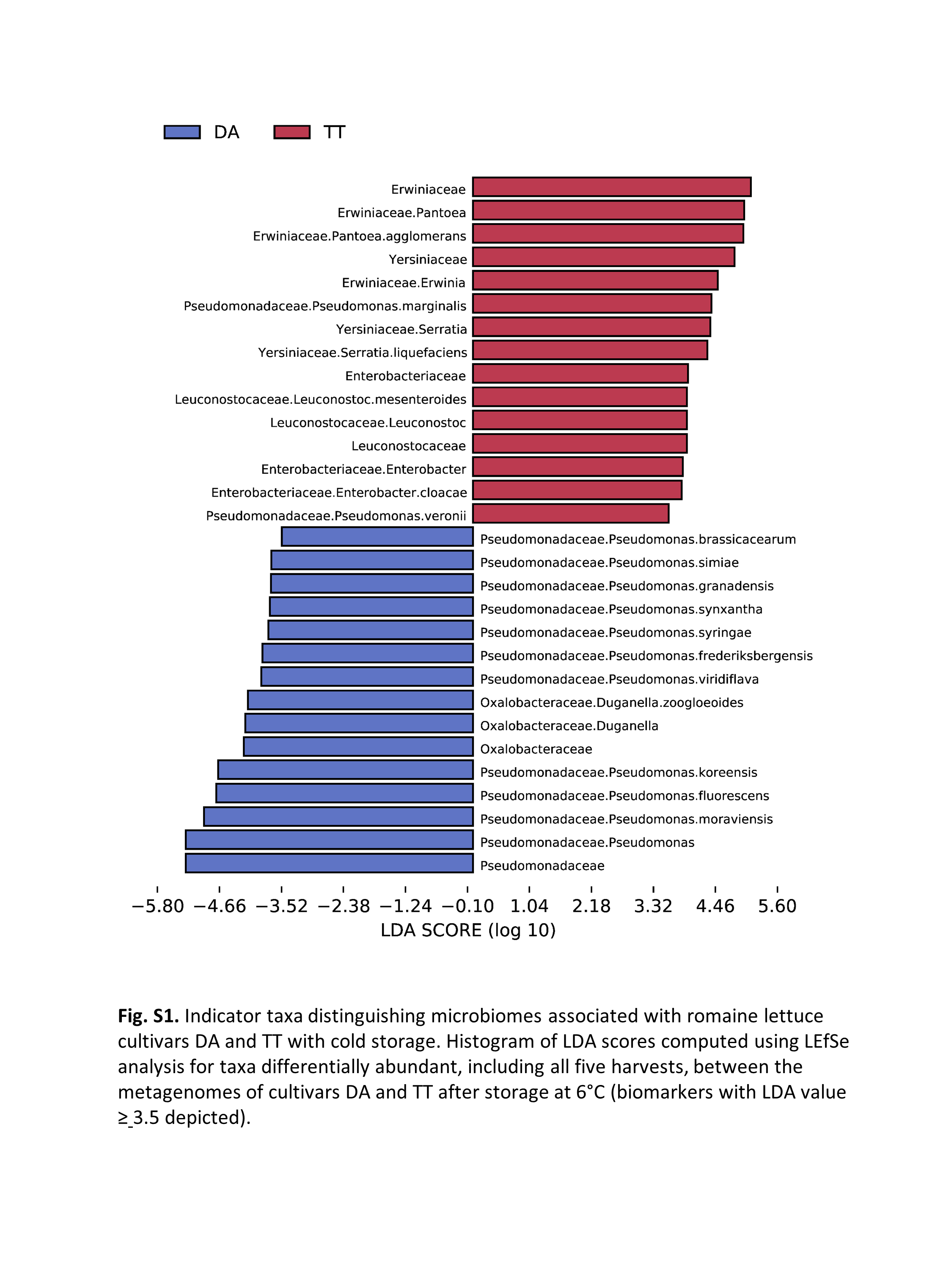

Supplement: Supplementary file 3 — Additional file 3: Fig. S1. Indicator taxa distinguishing microbiomes associated with romaine lettuce cultivars DA and TT with cold storage. Histogram of LDA scores computed using LEfSe analysis for taxa differentially abundant, including all five harvests, between the metagenomes of cultivars DA and TT after storage at 6°C (biomarkers with LDA value ≥ 3.5 depicted). [file 40793_2021_393_MOESM3_ESM.tif]

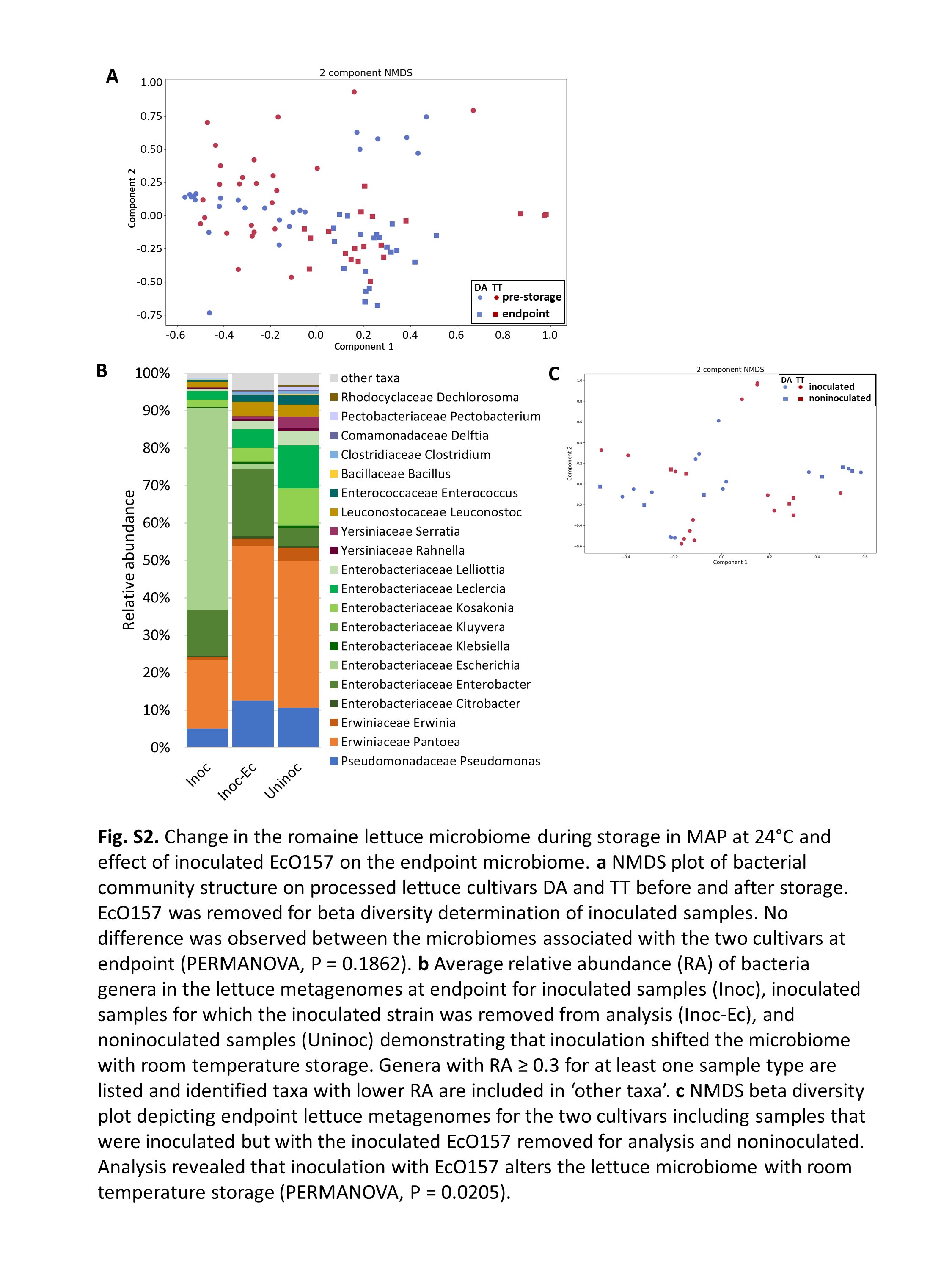

Supplement: Supplementary file 4 — Additional file 4: Fig. S2. Change in the lettuce microbiome during storage at 24°C in MAP as shown by (A) NMDS plot of TT and DA microbiome before and after storage, (B) relative abundance of bacteria genera on inoculated, inoculated (with EcO157 removed from microbiome analysis), and noninoculated samples, and (C) NMDS beta diversity plot revealing the effect of inoculated EcO157 on lettuce microbiome composition. [file 40793_2021_393_MOESM4_ESM.tif]

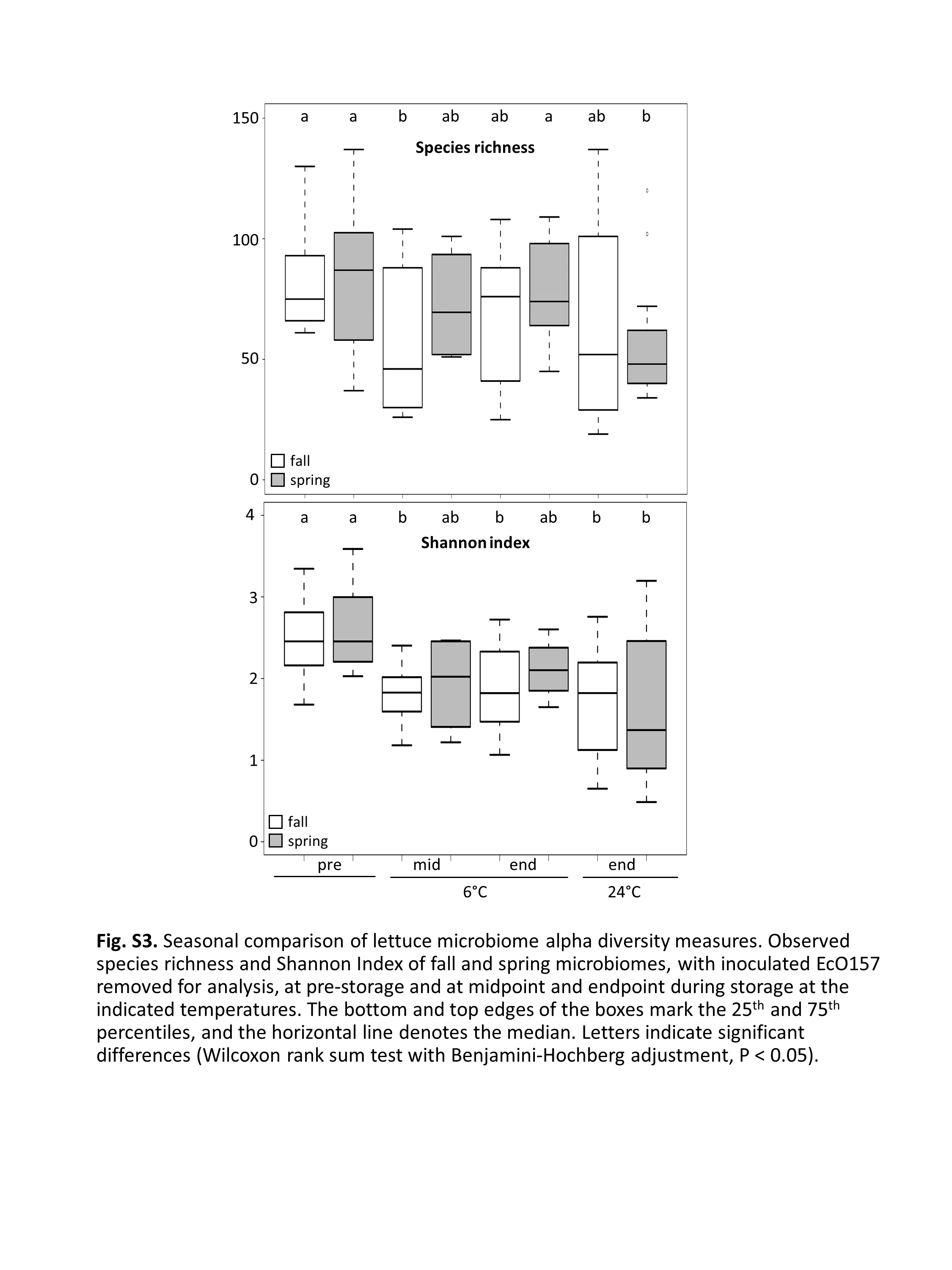

Supplement: Supplementary file 7 — Additional file 7: Fig. S3. Seasonal comparison of lettuce microbiome alpha diversity measures. Observed species richness and Shannon Index of fall and spring microbiomes, with inoculated EcO157 removed for analysis, at pre-storage and at midpoint and endpoint during storage at the indicated temperatures. The bottom and top edges of the boxes mark the 25th and 75th percentiles, and the horizontal line denotes the median. Letters indicate significant differences (Wilcoxon rank sum test with Benjamini-Hochberg adjustment, P < 0.05). [file 40793_2021_393_MOESM7_ESM.tif]

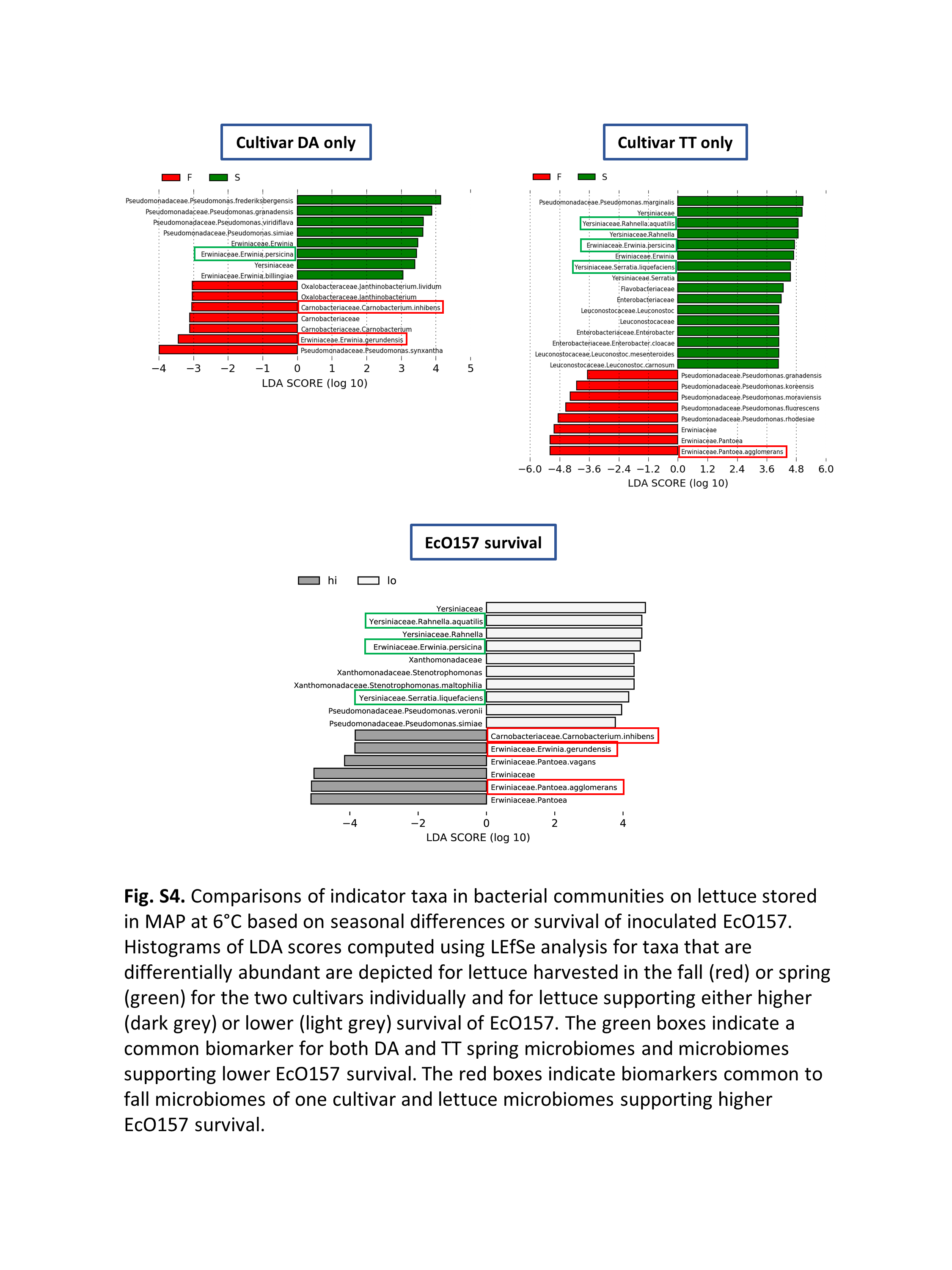

Supplement: Supplementary file 9 — Additional file 9: Fig. S4. Comparisons of indicator taxa in bacterial communities on lettuce stored in MAP at 6°C based on seasonal differences or survival of inoculated EcO157. Histograms of LDA scores computed using LEfSe analysis for taxa that are differentially abundant are depicted for lettuce harvested in the fall (red) or spring (green) for the two cultivars individually and for lettuce supporting either higher (dark grey) or lower (light grey) survival of EcO157. The green boxes indicate a common biomarker for both DA and TT spring microbiomes and microbiomes supporting lower EcO157 survival. The red boxes indicate biomarkers common to fall microbiomes of one cultivar and lettuce microbiomes supporting higher EcO157 survival. [file 40793_2021_393_MOESM9_ESM.tif]

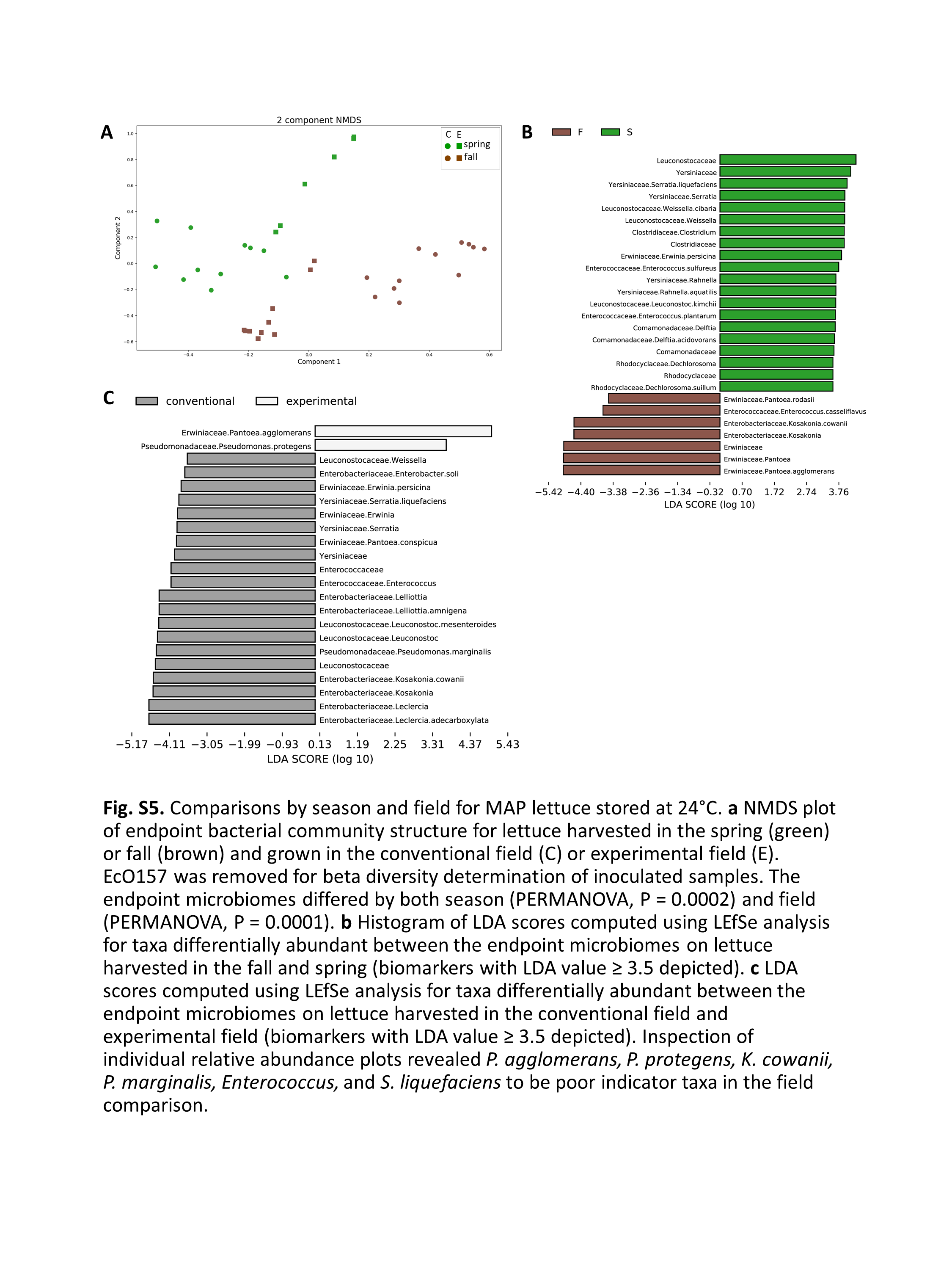

Supplement: Supplementary file 10 — Additional file 10: Fig. S5. Comparisons by season and field for MAP lettuce stored at 24°C. a NMDS plot of endpoint bacterial community structure for lettuce harvested in the spring (green) or fall (brown) and grown in the conventional field (C) or experimental field (E). EcO157 was removed for beta diversity determination of inoculated samples. The endpoint microbiomes differed by both season (PERMANOVA, P = 0.0002) and field (PERMANOVA, P = 0.0001). b Histogram of LDA scores computed using LEfSe analysis for taxa differentially abundant between the endpoint microbiomes on lettuce harvested in the fall and spring (biomarkers with LDA value ≥ 3.5 depicted). c LDA scores computed using LEfSe analysis for taxa differentially abundant between the endpoint microbiomes on lettuce harvested in the conventional field and experimental field (biomarkers with LDA value ≥ 3.5 depicted). Inspection of individual relative abundance plots revealed P. agglomerans, P. protegens, K. cowanii, P. marginalis, Enterococcus, and S. liquefaciens to be poor indicator taxa in the field comparison. [file 40793_2021_393_MOESM10_ESM.tif]
